# Predicting medication non-adherence using machine learning: Incorporating Complementary and Alternative Medicine (CAM) beliefs in Malaysian chronic disease patients

**DOI:** 10.1371/journal.pone.0354682

**Published:** 2026-07-30

**Authors:** Firdaus Aziz, Sorayya Malek, Ahmad Firdhaus Arham, Mashitoh Yaacob, Putri Nur Fatin Amir Rudin, Paik Ling Chuah, Adliah Mhd Ali

**Affiliations:** 1 Pusat Pengajian Citra Universiti, Universiti Kebangsaan Malaysia, Bandar Baru Bangi, Selangor, Malaysia; 2 Bioinformatics Science Programme, Institute of Biological Sciences, Universiti Malaya, Kuala Lumpur, Malaysia; 3 Centre for Quality Management of Medicines (QMM), Faculty of Pharmacy, Universiti Kebangsaan Malaysia, Kuala Lumpur, Malaysia; 4 Pharmacy Department, Hospital Kuala Lumpur, Malaysia; Korea University - Seoul Campus: Korea University, KOREA, REPUBLIC OF

## Abstract

Medication non-adherence among chronic disease patients remains a major contributor to poor health outcomes and medication wastage, particularly in multi-ethnic populations such as Malaysia where cultural and religious beliefs strongly influence health behaviours. This study aimed to develop and evaluate machine learning models that integrate demographic, clinical, and complementary and alternative medicine (CAM) belief factors to predict medication non-adherence among patients with type 2 diabetes mellitus, hypertension, and dyslipidaemia. A cross-sectional survey was conducted using a structured questionnaire comprising demographic and clinical data, history of CAM use, the 17-item Complementary and Alternative Medicine Beliefs Inventory (CAMBI), and the Malaysian Medication Adherence Scale (MALMAS). Twelve conventional machine learning algorithms and three stacked ensemble models were utilised using both balanced and unbalanced datasets with all variables as well as feature-selected variables. The best-performing model was a stacked ensemble using logistic regression-selected variables with the unbalanced dataset, achieving the highest AUC of 0.816. Feature selection identified significant variables including CAM beliefs (natural and holistic), race, number of daily doses, number of medications prescribed, religion, educational level, treatment duration, and hypertension status which were later interpreted using SHapley Additive exPlanations (SHAP) analysis. Model performance was further evaluated using the Youden Index and Decision Curve Analysis (DCA) to stratify patients into lower- and higher-risk groups, with a suitable cutoff identified at 0.4. These findings show that incorporating cultural and belief-related factors into machine learning models provides a novel, population-specific approach to predict better non-adherence and guide targeted interventions to reduce medication wastage in chronic disease management.

## Introduction

The Asia-Pacific region is home to a significant proportion of the global population suffering from chronic diseases, particularly diabetes, hypertension, and dyslipidaemia. These conditions are rising at an alarming pace, with diabetes alone projected to affect millions more in the coming decades [1]. In Malaysia, this growing burden is underscored by high adult prevalence rates of diabetes (18.3%), hypertension (30.0%), and hypercholesterolemia (47.7%) [2]. Managing these conditions requires strict medication adherence, defined by the World Health Organization (WHO) as “the degree to which the person’s behaviour corresponds with the agreed recommendations from a healthcare provider” [3]. However, an estimated 50% of chronic disease patients fail to adhere to their prescribed regimens [4], leading to poor disease control and increased mortality. In Malaysia, this issue is compounded by the widespread use of Complementary and Alternative Medicine (CAM) [5].

The integration of CAM is not merely a preference but a profound reflection of the cultural and religious fabric defining healthcare decisions in Asia. With nearly 70% of Malaysian adults engaging in CAM [6], patients frequently perceive traditional remedies as natural, safe, and spiritually congruent alternatives to conventional pharmacotherapy [7]. This creates a significant clinical paradox where while conventional medications remain essential for preventing severe disease progression [8], deeply rooted holistic beliefs often erode a patient’s perceived necessity for these treatments. Despite this critical tension, existing adherence research frequently treats conventional medicine and CAM in isolation, leaving a critical gap in understanding how culturally embedded health beliefs directly influence non-adherence behaviours.

Health psychology, particularly the Necessity-Concerns Framework, helps explain how alternative health beliefs can influence medication adherence. Strong beliefs in CAM efficacy may reduce patients’ perceived need for long-term pharmacotherapy while increasing concerns about the side effects of conventional medicines [9]. As a result, cultural beliefs in natural products and psychosocial factors may lead some patients to modify or discontinue prescribed treatments [10]. Although many CAM users practice dual use, studies consistently show that they are more likely to reduce or stop conventional medications across chronic conditions [11,12].

To address these multi-faceted behavioural complexities, recent studies have turned to machine learning, leveraging its capacity to decode high-dimensional data and non-linear interactions. However, most prior machine learning models have focused exclusively on clinical and demographic silos, ignoring the potent influence of CAM-related factors.

Therefore, the primary objective of this study is to develop and evaluate an advanced predictive model to identify the risk of medication non-adherence among Malaysian patients with chronic diseases. The main outcome of this study is the classification of a patient’s medication adherence status, determined via the Malaysian Medication Adherence Scale (MALMAS) [13]. To achieve this, the predictive framework integrates clinical data with psychosocial CAM beliefs, captured via the Complementary and Alternative Medicine Beliefs Inventory (CAMBI) [14]. Specifically, it is anticipated that patients holding stronger CAM beliefs, particularly the perception that natural holistic treatments are safer or superior, will demonstrate a significantly higher risk of non-adherence to their prescribed conventional medications. Furthermore, rather than relying on opaque algorithms, this study emphasises clinical relevance by utilising methodologies that ensure transparent model interpretability and rigorously evaluate real-world clinical utility, ultimately establishing actionable thresholds to guide patient risk stratification and reduce medication wastage in chronic diseases management.

## Methods

### Study participants

This cross-sectional study was conducted at the Medical Outpatient Clinic at one of the tertiary hospitals in Malaysia. A total of 270 patients were recruited over a five-month period from February 2017 to June 2017. The target population comprised individuals diagnosed with type 2 diabetes mellitus, hypertension, and/or dyslipidaemia. Participants were selected using a convenience sampling method. Inclusion criteria were as follows: patients aged 18 years and above at the time of recruitment; those under hospital follow-up for at least one of the aforementioned chronic medical conditions; individuals who had been on prescribed medication therapy for the condition(s) for a minimum of 12 months; and those able to read and/or understand either English or Malay. Exclusion criteria included pregnant individuals and those with psychotic disorders, cognitive impairments, or significant visual and/or hearing impairments.

This study received ethical approval from the Universiti Kebangsaan Malaysia Research Ethics Committee (Ethics Committee Approval Reference: UKM PPI/111/8/JEP-2017–057). Potential respondents at the outpatient clinic were invited to participate in the study. The researcher approached patients or their caregivers and explained the purpose of the study using the patient information leaflet. Patients who agreed to participate were provided with an explanatory statement and asked to sign an informed consent form. During the recruitment phase, approximately 332 individuals were approached, with 62 declining to participate primarily due to time constraints, resulting in the final sample of 270 participants. To ensure data integrity and quality control, a real-time verification process was implemented during data collection. Researchers reviewed each questionnaire immediately upon completion in the presence of the participant to identify and resolve any ambiguous responses or skipped items. This active oversight ensured that all 270 collected responses were complete cases, thereby eliminating the need for post-hoc data imputation due to missing data. Additionally, incomplete survey responses were omitted, as missing answers, particularly to key questions, could compromise data quality and lead to inaccurate conclusions. Individuals unable to understand either English or the native language were excluded from the survey.

### Informed consent

Potential respondents at the outpatient clinic were invited to participate in the study. The researcher approached patients or their caregivers and explained the purpose of the study using the patient information leaflet. Patients who agreed to participate were provided with an explanatory statement and asked to sign an informed consent form. Individuals unable to understand either English or the native language, were excluded from the survey.

### Study instrument

Data collection for this study was carried out using a set of validated questionnaires available in both English and Malay languages. For instruments that were originally in English and lacked a Malay version, particularly those assessing health belief factors, a standard forward-backward translation method was employed to ensure linguistic and conceptual equivalence. The structured questionnaires were administered via face-to-face interviews conducted by the research team. Researchers actively assisted participants in reading and completing the survey to ensure full comprehension of the items, minimise missing data, and accommodate elderly patients or those with visual impairments. The questionnaire comprised both categorical and continuous variables and was divided into four distinct sections.

Section A gathered information on participants’ demographic and clinical characteristics, including age, gender, race, religion, marital status, educational level, employment status, household income (per month), status of type 2 diabetes mellitus, hypertension, and/or dyslipidaemia, number of medications prescribed over the past 12 months, number of daily doses, duration of treatment (in months), and self-reported health status. Self-reported health status was adapted from the general health perception item of the SF-36 Health Survey. The SF-36 is a rigorously validated instrument demonstrating high test-retest reliability (r > 0.80) and strong construct validity across diverse populations [15]. This section provided essential baseline information for understanding the study population.

Section B focused on information regarding history of CAM use. In this study, CAM was defined as “a practice of medicine that is other than the practice of medicine or dental practices utilised by registered medical or dental practitioners” [16]. A CAM-user was considered to have used one or more of the CAM modalities for the stated medical disease in the past 12 months at the time of the study. This section comprised of a pre-set list of CAM modalities as described in the questionnaire. Vitamin and supplements were added as an extra variable to distinguish its use from herbal products. Lastly, an empty column was provided in this section to allow for patients to state any use of CAM which was not specified in the questionnaire.

Section C assessed health belief factors consisting of 17-item scale based on the Complementary and Alternative Medicine Beliefs Inventory (CAMBI) [14]. It measured three domains: – belief in natural treatment (Question 1–6), belief in participation in treatment (Question 7–11) and belief in holistic health (Question 12–17). The CAMBI survey has been previously validated and demonstrated satisfactory internal consistency. The reported Cronbach’s alpha for the overall scale was 0.81, with individual domain subscales also showing acceptable reliability where belief in natural treatment (α = 0.75), belief in participation in treatment (α = 0.68), and belief in holistic health (α = 0.73). Furthermore, reliability analysis conducted on this current study cohort confirmed the instrument’s robustness, yielding a highly satisfactory overall Cronbach’s alpha of 0.918. The internal consistency for the specific domains within this study’s sample was equally strong: belief in natural treatment (α = 0.895), belief in participation in treatment (α = 0.705), and belief in holistic health (α = 0.836). Each item in this section was scored on a seven-point Likert scale ranging from one (strongly disagree) to seven (strongly agree). After reversing the codes of the negatively worded items (item 9, 11, 14, 17), item scores were summed to provide the total sub-scaled scores of each domain, i.e., belief in natural treatment (ranged 1–42), belief in participation in treatment (ranged 1–35) and belief in holistic health (ranged 1–42). Median cut-offs were obtained for each sub-scale score. A median cut-off points of ≥32 suggests positive belief in natural treatment; a median cut-off point of ≥23 suggests positive belief in participation in treatment and a median cut-off point of ≥29 suggests positive belief in holistic health. Scores equal or less than these cut off points were considered negative belief.

The MALMAS is a rigorously validated instrument specifically designed for the Malaysian population, demonstrating high internal reliability with a reported Cronbach’s alpha of 0.88, alongside strong concurrent validity [13,17]. The first item of the MALMAS consisted of five responses: (1) All the time, (2) Often, (3) Sometimes, (4) Rarely and (5) Never. The remaining seven items consisted of dichotomous response of “Yes” or “No”. The responses in the MALMAS had a total score which ranged from 0 to 8. Patients were considered adherent if they had a total score of six and above (≥ 6), and non-adherent if they had a total score of below six (< 6) [13,17]. Medication adherence, as measured by the MALMAS, served as the primary outcome of this study and was used as the target variable for prediction.

### Data pre-processing

The study utilised a complete dataset of 270 responses, requiring no imputation, meaning there were no missing values in either the predictor variables or the outcome variable. Variables were recoded for numerical processing [18], and those exhibiting near-zero variance were removed to prevent instability [19]. Following this, 19 predictor variables (7 continuous and 12 categorical) were retained. Continuous variables were standardised using Min-Max Normalisation to prevent scale-driven bias [20].

To ensure robust evaluation and prevent data leakage, all preprocessing steps were performed independently on training and validation subsets [21]. The data was partitioned into a 70% training set (for model development) and a 30% untouched validation set (for evaluation) using stratified random sampling to maintain proportional representation of adherence groups. The validation set was kept completely separate during model training and was only used to assess final model performance, ensuring an unbiased evaluation of predictive accuracy.

To evaluate the impact of class imbalance, models were trained on both the original unbalanced dataset (17:13 ratio) and a balanced dataset generated via Synthetic Minority Oversampling Technique-Tomek Links (SMOTE-Tomek) [22]. SMOTE generates synthetic samples of the minority class (in this case, non-adherent patients) by interpolating between existing minority class example. Tomek links, on the other hand, help clean the dataset by identifying and removing ambiguous samples that are close to the decision boundary between classes. By combining both techniques, SMOTE-Tomek not only balances the class distribution but also improves the overall quality of the training data, enhancing the model’s ability to generalise and reducing the likelihood of overfitting to noise. Model stability was ensured through stratified 5-fold cross-validation, with hyperparameters optimised via grid search. Specific hyperparameter configurations are detailed in S1 Table.

### Feature selection

Feature selection plays a crucial role in reducing data dimensionality and simplifying model complexity, ultimately leading to better model performance and interpretability. By identifying and retaining only the most relevant variables, this process offers several advantages such as, improved prediction accuracy, reduced risk of overfitting, enhanced model transparency, lower storage requirements, and importantly, decreased time and cost associated with administering questionnaires during clinical consultations [23].

In this study, the sequential backward elimination technique was employed to identify the most significant predictors. This method begins with the full set of input variables and iteratively removes those that contribute the least to model performance. The process is repeated until no further variables can be eliminated without negatively affecting model accuracy (or in this study, Area Under the Receiver Operating Characteristic Curve (AUC) values was use as the determinant), resulting in a leaner and more efficient feature set. From the original 19 variables this method helped to eliminate irrelevant or redundant variables while preserving predictive strength [24].

Three machine learning algorithms, Random Forest (RF), Logistic Regression (LR), and Support Vector Machine (SVM) were used to guide the feature selection process. Initially, models were trained using the complete set of variables, and feature importance scores were generated. Variables were then ranked based on these scores, and the least important variable determined by the lowest contribution to the AUC was removed. The model was retrained using the reduced set of variables, and performance was re-evaluated using AUC. This cycle of elimination and retraining continued until no substantial improvement in model performance was observed compared to the previous iteration. The final subset of variables retained through this process was considered the optimal feature set and was used in subsequent stages for model training, evaluation, and interpretation.

### Model development

Following data pre-processing and feature selection, predictive models were developed using both the original unbalanced dataset and a balanced dataset generated via SMOTE-Tomek. Models were trained using two feature sets: the full 19-variable set and subsets identified through sequential backward elimination. To develop predictive models for medication non-adherence, this study employed twelve machine learning algorithms comprising linear, non-linear, probabilistic, and tree-based methods. Linear models included Logistic Regression (LR) [25] and Support Vector Machine (SVM) with a linear kernel [26]. Non-linear and distance-based methods included SVM with a radial basis function (RBF) kernel [26], k-Nearest Neighbours (k-NN) [27], and Gaussian Process [28]. Probabilistic approaches consisted of Gaussian Naïve Bayes [29] and Bernoulli Naïve Bayes [30], while tree-based and ensemble methods included Decision Tree [31], Random Forest (RF) [32], AdaBoost [33], Gradient Boosting [34], and Bagging [35]. These algorithms were selected to capture diverse learning paradigms and predictive capabilities for medication non-adherence classification.

To further improve predictive performance and model robustness, stacked ensemble learning was implemented by combining the outputs of all twelve base learners through a meta-learning framework [36]. Stacked ensemble learning enables the integration of heterogeneous models to reduce generalisation error, mitigate overfitting, and improve prediction stability and accuracy [37]. Three stacked ensemble configurations were developed using Generalised Linear Model (GLM) [37], Random Forest (RF) [38], and Gradient Boosting Machine (GBM) [39] as meta-learners. The Ensemble GLM prioritised interpretability, Ensemble RF emphasised robustness and variance reduction, while Ensemble GBM focused on iterative optimisation and predictive accuracy. The systematic combination of these various data balancing techniques, feature subsets, algorithm families, and ensemble strategies ultimately yielded 120 distinct model configurations. Evaluating this extensive array of models served as a sensitivity analysis, ensuring that the final selection was robust, stable, and not overly dependent on a single methodological approach.

### Model evaluation, validation, and performance measures

Model evaluation and validation were conducted using descriptive statistics, statistical hypothesis testing, and standard machine learning performance metrics. Frequencies were reported for categorical variables, while means and standard deviations were used for continuous variables. Univariate analysis was performed using chi-square tests for categorical variables and two-sided independent Student’s t-tests for continuous variables, with significance set at p < 0.001. This stringent threshold was specifically selected to control for multiple testing across the 19 predictor variables and to reduce the risk of Type I errors, ensuring that only variables with a highly robust statistical association were prioritised for the subsequent machine learning feature selection process.

Model performance was evaluated using area under the receiver operating characteristic curve (AUC), balanced accuracy, sensitivity, specificity, positive predictive value (PPV), and negative predictive value (NPV). AUC was selected as the primary metric due to its robustness in handling imbalanced datasets. To statistically compare model performance, a paired corrected resampled t-test was applied to account for dependencies arising from repeated training-validation splits.

Following identification of the best-performing model, SHapley Additive exPlanations (SHAP) [40] were used to interpret variable contributions toward medication non-adherence prediction. Variables were ranked using mean absolute SHAP values and visualised using SHAP feature importance and beeswarm plots to illustrate the direction and magnitude of each variable’s effect on prediction outcomes.

To determine the optimal classification threshold, both the Youden Index [41] and Decision Curve Analysis (DCA) [42] were employed. The Youden Index was used to identify thresholds that maximised sensitivity and specificity, while DCA evaluated the net clinical benefit across varying probability thresholds [43,44]. In addition, model calibration was assessed using Platt scaling, with comparisons between original and calibrated models performed using log loss, Brier score, χ² goodness-of-fit, and associated *p-values*. All analyses, including preprocessing, statistical testing, model evaluation, calibration, and SHAP analysis, were conducted using Python libraries including *scikit-learn*, *scipy*, and *numpy*.

**Fig 1:**
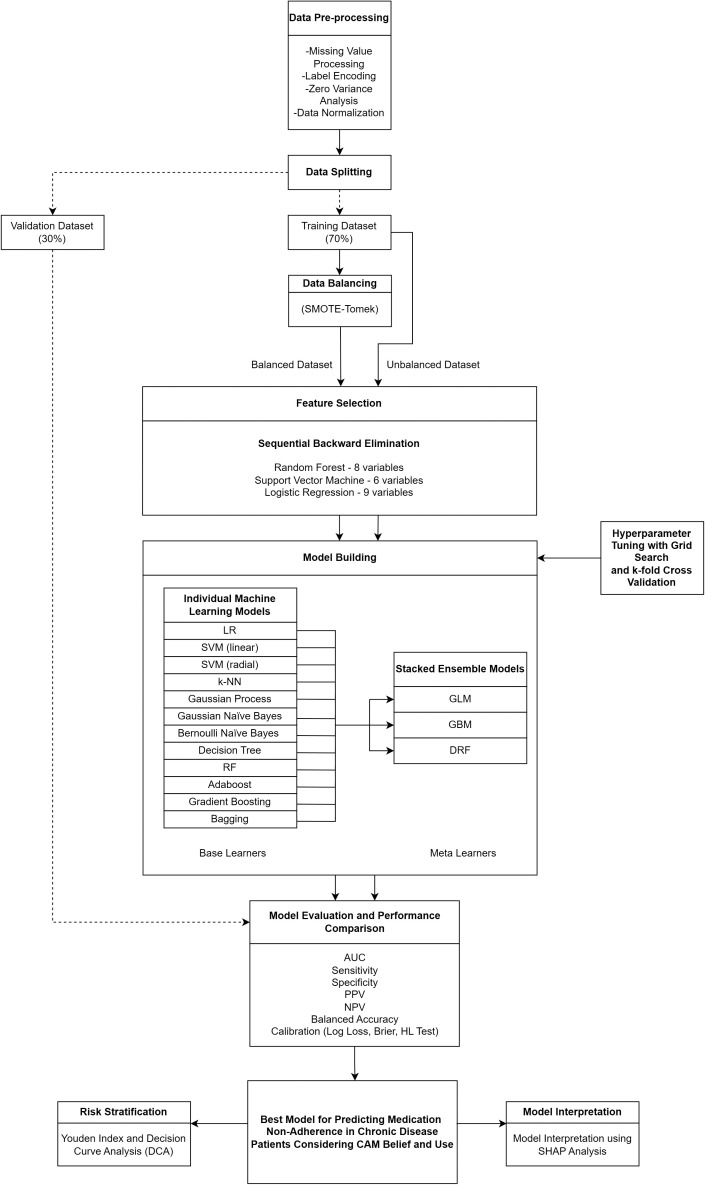
Overall methodological flow of the study.

Together, these methods ensured a balance between predictive accuracy and practical clinical relevance, supporting informed decision-making based on the model’s output. The complete workflow of this process is summarised in the flowchart in Fig 1.

## Results

A total of 270 patients were analysed, with 153 (56.7%) classified as adherent and 117 (43.3%) as non-adherents. The study population was predominantly female (60.0%) with a mean age of 56.4 years (SD ± 12.3). The clinical profile was characterised by high rates of dyslipidaemia (77.0%), diabetes (71.1%), and hypertension (65.2%). Additionally, 53.7% of participants reported using complementary and alternative medicine (CAM).

Statistical analysis revealed several key differences between the groups (*p* < 0.001):

- Treatment Burden: Non-adherent patients managed a higher mean number of medications (4.5 vs 2.9) and daily doses (6.4 vs 3.9) compared to adherent patients.
- Clinical Status: Significant associations were found between adherence and the presence of diabetes, hypertension, and dyslipidaemia.
- Health Perception & Beliefs: Non-adherent patients reported poorer self-reported health status and demonstrated stronger beliefs in natural treatment, holistic health, and participation in treatment.
- CAM Engagement: A greater tendency to use CAM was observed in the non-adherent group.

These findings, detailed in Table 1, suggest that adherence is significantly influenced by treatment complexity, perceived disease burden, and health-related beliefs.

**Table 1:** Statistical analysis of each variable in the dataset.

Variables	Attributes	Total	Adherent	Non-adherent	p-value
N		270	153 (56.7)	117 (43.3)	
Age		56.4 ± 12.3	55.2 ± 12.8	57.9 ± 11.3	0.069
Gender	Male	108 (40.0)	62 (39.3)	46 (40.5)	0.841
	Female	162 (60.0)	71 (60.7)	91 (59.5)	
Race	Malay	141 (52.2)	76 (49.7)	65 (55.6)	0.489
	Chinese	88 (32.6)	53 (34.6)	35 (29.9)	
	Indian	39 (14.4)	22 (14.4)	17 (14.5)	
	Others	2 (0.7)	2 (1.3)	0 (0.0)	
Religion	Islam	142 (52.6)	77 (50.3)	65 (55.6)	0.632
	Buddhist	72 (26.7)	44 (28.8)	28 (23.9)	
	Hindu	26 (9.6)	15 (9.8)	11 (9.4)	
	Christian	28 (10.4)	15 (9.8)	13 (11.1)	
	Others	2 (0.7)	2 (1.3)	0 (0)	
Marital status	Single	49 (18.1)	33 (21.6)	16 (13.7)	0.245
	Married	217 (80.4)	118 (77.1)	99 (84.6)	
	Divorced	4 (1.5)	2 (1.3)	2 (1.7)	
Educational level	Primary	55 (20.4)	26 (17.0)	29 (24.8)	0.163
	Secondary	145 (53.7)	82 (53.6)	63 (53.8)	
	Tertiary	70 (25.9)	45 (29.4)	25 (21.4)	
Employment status	Unemployed	120 (44.4)	64 (41.8)	56 (47.9)	0.551
	Employed	116 (43.0)	70 (45.8)	46 (39.3)	
	Self-employed	34 (12.6)	19 (12.4)	15 (12.8)	
Household income (per month)	<MYR1000	41 (15.2)	26 (17.0)	15 (12.8)	0.899
	MYR1000–1999	41 (15.2)	22 (14.4)	19 (16.2)	
	MYR2000–2999	45 (16.7)	26 (17.0)	19 (42.2)	
	MYR3000–3999	47 (17.4)	28 (18.3)	19 (40.4)	
	MYR4000–4999	50 (18.5)	27 (17.6)	23 (19.7)	
	>MYR5000	46 (17.0)	24 (15.7)	22 (18.8)	
Diabetes status	Yes	192 (71.1)	87 (56.9)	105 (89.7)	**p < 0.001**
	No	78 (28.9)	66 (43.1)	12 (10.3)	
Hypertension status	Yes	176 (65.2)	87 (56.9)	89 (76.1)	**p < 0.001**
	No	94 (34.8)	66 (43.1)	28 (23.9)	
Dyslipidaemia status	Yes	208 (77.0)	107 (69.9)	101 (86.3)	**p < 0.001**
	No	62 (23.0)	46 (30.1)	16 (13.7)	
Number of medications prescribed		3.6 ± 1.9	2.9 ± 1.8	4.5 ± 1.8	**p < 0.001**
Number of daily doses of medications		5.0 ± 3.0	3.9 ± 2.7	6.4 ± 2.7	**p < 0.001**
Duration of treatment (months)		98.8 ± 86.0	91.7 ± 91.9	108.0 ± 76.9	0.122
Self-reported health status	Excellent	7 (2.6)	6 (3.9)	1 (0.9)	**p < 0.001**
	Very good	82 (30.4)	65 (42.5)	17 (14.5)	
	Good	114 (42.2)	55 (35.9)	59 (50.4)	
	Fair	60 (22.2)	23 (15.0)	37 (31.6)	
	Poor	7 (2.6)	4 (2.6)	3 (2.6)	
CAM user	Yes	145 (53.7)	52 (34.0)	93 (79.5)	**p < 0.001**
	No	125 (46.3)	101 (66.0)	24 (20.5)	
Belief in natural treatment		31.9 ± 6.3	29.4 ± 5.7	35.1 ± 5.5	**p < 0.001**
Belief in participation in treatment		23.2 ± 2.8	22.5 ± 2.6	24.1 ± 2.7	**p < 0.001**
Belief in holistic health		29.4 ± 5.1	27.8 ± 4.6	31.5 ± 5.0	**p < 0.001**

p-value is statistically significant as **p < 0.001**.

Data are expressed as count (percentage), or mean ± standard deviation, as appropriate.

### Feature selection

To identify the most significant variables for predicting medication non-adherence among patients with chronic diseases, sequential backward elimination approach was applied. This method was conducted using three different machine learning algorithms, RF, SVM and LR to ensure robustness in feature selection and the selected variables are as seen on Table 2 below. Through this process, three variables consistently emerged as important across all three methods: race, hypertension status, and belief in natural treatment. These variables represent the common set of predictors most strongly associated with medication non-adherence. Subsequently, predictive models were constructed in two ways: first, using the full set of available variables (19 variables), and second, using only the subset of variables identified by the sequential backward elimination approach (RF-selected = 8 variables, SVM-selected = 6 variables and LR-selected = 9 variables) as seen in Table 2 below. This comparison allowed us to evaluate whether models trained on a reduced, more targeted feature set could achieve comparable or improved predictive performance relative to those trained on the complete feature set.

**Table 2:** Variables selected using sequential backward elimination with three different machine learning algorithms; SVM, RF, and LR.

SVM-selected variables (6 variables)	RF-selected variables (8 variables)	LR-selected variables (9 variables)
**Race**	Gender	**Race**
Religion	**Race**	Religion
Educational Level	Diabetes status	Educational level
**Hypertension status**	**Hypertension status**	**Hypertension status**
Number of daily doses of medications	CAM user	Number of medications prescribed
**Belief in natural treatment**	Number of medications prescribed	Number of daily doses of medications
	Duration of treatment	Duration of treatment
	**Belief in natural treatment**	**Belief in natural treatment**
		Belief in holistic health

The common variables are in bold

### Model performance

To comprehensively evaluate the models’ performances, a wide range of model configurations were developed and assessed in this study. The models were constructed using different data settings, including both balanced and unbalanced datasets, and were trained on two types of feature sets: (i) the full set of variables and (ii) the selected sets of variables selected through sequential backward elimination using RF, SVM and LR. A total of 120 predictive models were evaluated across various data and feature configurations (full results available in S2 Table). Table 3 presents the best-performing models for each combination of dataset setting and feature selection method.

**Table 3:** Best performing models from each combination of dataset setting and feature selection method.

Feature Selection & Dataset	Best Models	Best AUC (95% CI)	Balanced Accuracy
All Variables (Balanced)	Gaussian NB	0.791 (0.702 - 0.879)	0.780
SVM Variables (Balanced)	k-NN	0.788 (0.699 - 0.877)	0.763
LR Variables (Balanced)	Ensemble RF	0.781 (0.691 - 0.871)	0.777
RF Variables (Balanced)	Gaussian NB	0.775 (0.684 - 0.866)	0.748
SVM Variables (Unbalanced)	Ensemble GLM	0.816 (0.732 - 0.901)	0.781
LR Variables (Unbalanced)	Ensemble GLM	0.816 (0.731 - 0.900)	0.785
RF Variables (Unbalanced)	Gaussian NB	0.793 (0.705 - 0.881)	0.731
All Variables (Unbalanced)	Gaussian NB	0.812 (0.727 - 0.897)	0.803

Overall, the top models demonstrated strong predictive capabilities, with Area Under the Curve (AUC) values ranging from 0.775 to 0.816. Key findings from the performance evaluation include:

- Top Individual Model: Gaussian Naïve Bayes (all variables, unbalanced dataset) achieved the highest performance among individual algorithms with an AUC of 0.812 evaluated on the 30% untouched validation dataset, significantly outperforming other individual configurations (*p* < 0.001).
- Top Ensemble Model: The Ensemble GLM trained on LR-selected variables with the unbalanced dataset achieved the highest overall AUC of 0.816 evaluated on the 30% untouched validation dataset. While it matched the AUC of the Ensemble GLM trained on SVM-selected variables, it was designated as the overall best-performing model due to a higher balanced accuracy (0.785 vs. 0.781). Differences in performance among the top ensemble models were not statistically significant (*p* > 0.001).

The overall best-performing model was derived from the original unbalanced dataset utilising targeted variable selection (LR), rather than all available variables or synthetic balancing. This suggests that targeted feature selection effectively reduced noise and optimised model learning, yielding superior performance over data balancing techniques in this cohort.

#### Model interpretation

**Fig 2:**
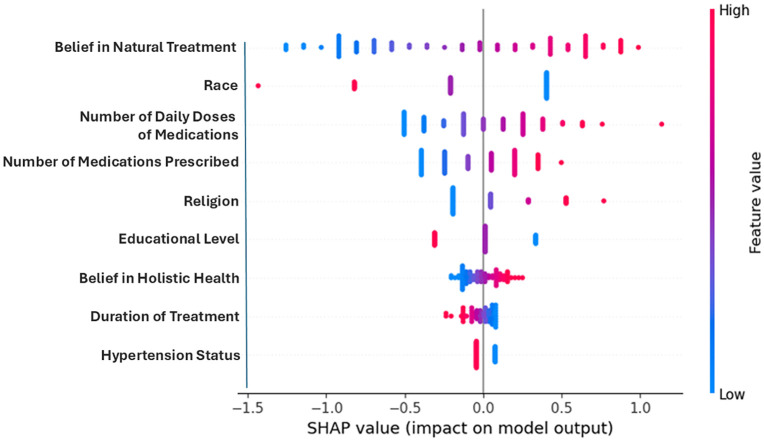
SHAP summary plot of the best performing model.

SHAP analysis was performed on the optimal model (Ensemble GLM trained on LR-selected variables, unbalanced dataset) to interpret the influence of each predictor. Fig 2 presents the SHAP beeswarm plot, which ranks the nine selected variables by their overall importance to the model’s predictions. Positive SHAP values indicate a higher likelihood of predicting medication non-adherence.

The directional impacts of these nine predictors are summarised as follows:

1. Belief in Natural Treatment: The most influential variable where stronger positive beliefs in natural remedies were significantly associated with a higher likelihood of non-adherence.
2. Race: Indigenous patients demonstrated a stronger tendency to adhere to prescribed treatments compared to other racial groups.
3. Treatment Burden: Higher numbers of prescribed daily doses (3rd) and total medications (4th) directly increased the probability of non-adherence, underscoring the negative impact of treatment complexity.
4. Religion: Identified as the fifth most important predictor contributing to the model’s decision-making process.
5. Educational Level: Higher educational attainment demonstrated a modest protective effect, associating with greater medication adherence.
6. Belief in Holistic Health: Similar to natural treatments, stronger positive attitudes toward holistic health practices were linked to increased non-adherence.
7. Duration of Treatment: Patients with longer treatment histories exhibited higher adherence rates compared to those at earlier stages of treatment.
8. Hypertension Status: A diagnosis of hypertension was slightly associated with a greater tendency to adhere compared to non-hypertensive patients.

### Risk stratification

**Fig 3:**
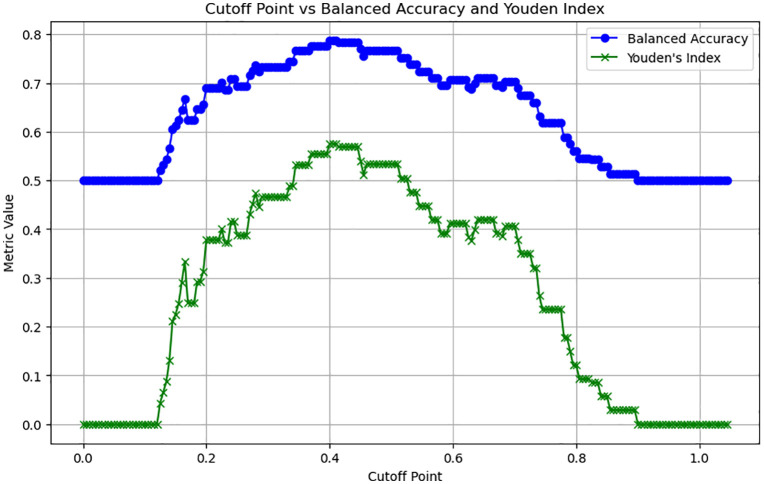
Graph of different cutoff points versus balanced accuracy of the Youden Index.

To establish the optimal classification threshold for clinical use, the Youden Index was evaluated. The Youden Index is a metric that balances sensitivity and specificity to identify the threshold that maximises the model’s overall discriminative ability, ensuring the best possible trade-off between correctly identifying non-adherent patients and minimising false positives. As illustrated in Fig 3, the balanced accuracy curve (blue line) and the Youden Index curve (green line) both demonstrated clear peaks within the 0.4 to 0.5 probability range. Specifically, a cutoff threshold of 0.4 was identified as optimal, yielding the highest balanced accuracy of 0.788 and a Youden Index of 0.576, alongside a sensitivity of 0.804 and specificity of 0.771. This threshold of 0.4 was selected as the final cutoff point, as it reflects the model’s ability to maintain strong diagnostic accuracy while minimising classification bias toward either adherence or non-adherence.

**Fig 4:**
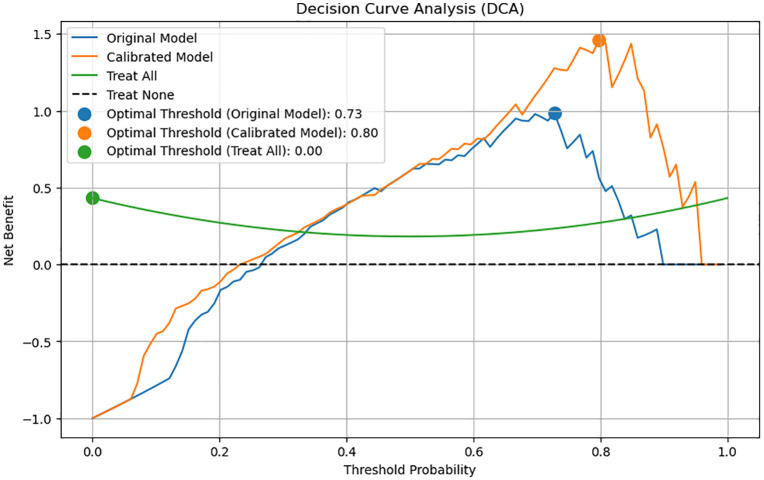
Decision Curve Analysis (DCA) of the original model (best performing model) and calibrated model.

To further evaluate clinical usefulness, Decision Curve Analysis (DCA) was employed. Unlike the Youden Index, DCA assesses the net clinical benefit across a continuous range of threshold probabilities, demonstrating whether the model adds practical value compared to default “treat all” or “treat none” strategies. In Fig 4, the x-axis represents the threshold probability for classifying a patient as non-adherent (triggering intervention), while the y-axis reflects the net benefit, which balances the gains of correctly identifying non-adherence (true positives) against the harms of unnecessary intervention (false positives).

The analysis revealed that the model calibrated via Platt Scaling (orange line) consistently demonstrated a higher net benefit than the original model (blue line) across most thresholds. The original model achieved its maximum net benefit of 0.98 at a threshold of approximately 0.73, whereas the calibrated model shifted to a threshold of 0.80 with a superior net benefit of 1.46. Both models significantly outperformed the “treat all” strategy (green line), which maintained a low baseline net benefit of 0.43, confirming that indiscriminate intervention is suboptimal. As expected, model performance converged with the “treat all” approach at near-zero thresholds and declined toward zero at extremely high thresholds due to missed cases. Ultimately, the DCA confirms that applying the calibrated model, particularly at the 0.80 threshold, provides the greatest overall clinical utility for guiding targeted patient interventions.

**Fig 5:**
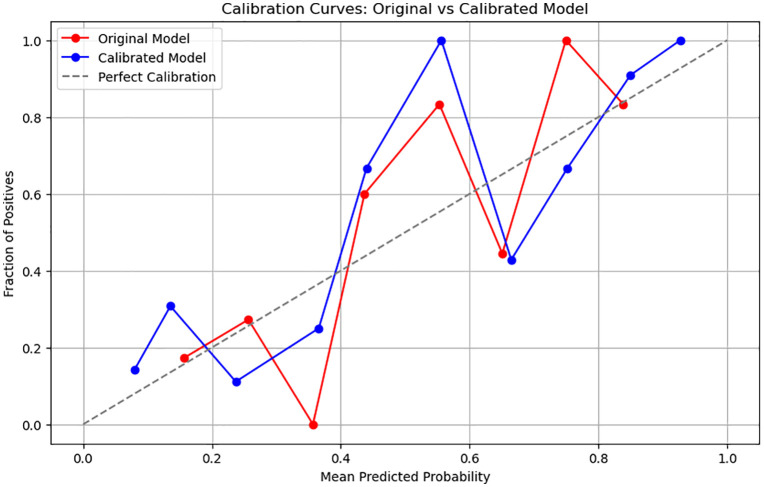
Calibration curve between original and calibrated model.

To further evaluate the robustness of the prediction model, a comparison between the original and calibrated models was conducted using calibration curve analysis (Fig 5) and several calibration metrics. As can be seen from Table 4, both models achieved the same AUC (0.816), indicating comparable discriminatory ability. However, the original model demonstrated slightly better probability estimation, with lower Log Loss (0.52 vs. 0.528) and Brier Score (0.17 vs. 0.171). In terms of calibration, the original model also performed better, as reflected by its lower *X*² statistic (6.407 vs. 11.44) and higher p-value (0.602 vs. 0.178), suggesting closer alignment to the ideal calibration line. These findings indicate that, despite calibration, the original model maintained more reliable predicted probabilities and overall performance. While the differences are small, the original model appears more suitable for clinical application.

**Table 4:** Calibration comparison measures between original and calibrated models.

Model	AUC	Log Loss	Brier score	X2 Goodness of fit	p-value
Original Model	0.816	0.520	0.170	6.407	0.602
Calibrated Model	0.816	0.528	0.171	11.44	0.178

To complement the calibration and DCA results, this study also compared the optimal cutoff values derived from the Youden Index and DCA as seen in Table 5 below. Both cutoffs yielded the same AUC of 0.816 (95% CI: 0.731–0.900), confirming that the model’s overall discriminatory ability was unaffected by the choice of threshold. However, the performance profiles differed. At a cutoff of 0.4, the model achieved a more balanced sensitivity (80.4%) and specificity (77.1%), resulting in higher balanced accuracy and Youden’s Index. In contrast, the higher cutoff of 0.73 led to markedly increased sensitivity (97.8%) but much lower specificity (34.3%), favouring the identification of nearly all non-adherent patients at the expense of more false positives. While the Youden Index pointed to 0.4 as the statistically optimal threshold and the DCA suggested benefit at higher cutoffs, this study aimed to identify the most suitable balance between false negatives and false positives. Therefore, both indices were considered and selected 0.4 as the final cutoff, with further justification provided in the discussion part.

**Table 5:** Comparison between Youden index and DCA.

Cutoff	Testing CV AUC	Testing Sensitivity	Testing Specificity	Balanced Accuracy	Youden Index
0.4	0.816 (0.731 - 0.900)	0.804	0.771	0.788	0.576
0.73	0.816 (0.731 - 0.900)	0.978	0.343	0.661	0.321

## Discussion

This study pioneers the application of machine learning to predict medication non-adherence in chronic disease patients by uniquely integrating Complementary and Alternative Medicine (CAM) beliefs. The key findings demonstrate that: (i) model performance was significantly enhanced by utilising sequential backward elimination for targeted feature selection; (ii) the stacked Ensemble GLM (trained on the unbalanced dataset with LR-selected variables) emerged as the optimal predictive model, achieving an AUC of 0.816; (iii) SHAP analysis successfully decoded the model’s decision-making, identifying belief in natural treatments, race, pill burden, and educational level among the most critical predictors of non-adherence; and (iv) risk stratification identified 0.40 as the clinically optimal classification threshold, balancing diagnostic accuracy with real-world utility. Ultimately, this framework establishes that machine learning can effectively predict adherence risk while providing actionable insights into complex behavioural determinants.

Existing literature evaluating the association between CAM use and medication non-adherence remains limited, often constrained to specific chronic diseases [45] or elderly populations [46], with only one study examining all three conditions included in this research [47]. Furthermore, previous studies predominantly focus on binary CAM use rather than nuanced patient beliefs and rely on conventional statistical methods that fail to capture complex, non-linear relationships [45]. Despite the wide heterogeneity of CAM practices, extensive research demonstrates that the broad operationalisation of CAM use as a binary variable (user vs. non-user) remains a highly significant predictor of non-adherence [48]. This predictive validity exists because the underlying belief system associated with CAM adoption, such as a preference for natural treatments, a desire for autonomy, and concerns regarding pharmaceutical side effects, is ultimately the core psychological driver of medication modification, proving more influential in shaping behavioural changes than the specific physical modality chosen [49]. Therefore, in predictive modelling, capturing these nuanced, shared psychological precursors provides a more accurate assessment of non-adherence risk than a granular categorisation of individual CAM practices.”

Crucially, there is a lack of predictive modelling utilising population-specific, multi-ethnic data. This study overcomes these limitations by employing advanced machine learning models tailored to the Asian Pacific and Malaysian context. Evaluating these variables is particularly warranted here, as the country’s multi-ethnic composition and diverse religious interpretations of healing exert a strong influence on health-related behaviours [50]. While CAM is most often employed in a complementary rather than substitutive manner, associated beliefs significantly influence necessity-concern evaluations, perceptions of side effects, and treatment expectations, thereby impacting adherence behaviours [51]. Given these complex, population-specific dynamics, the integration of CAM beliefs and usage into adherence prediction models is not only justified but essential for accurately capturing determinants of medication-taking behaviour in Malaysia and across the Asian Pacific and indirectly mitigate the issue of medication wastage.

A questionnaire-based design was utilised as it is particularly advantageous when exploring belief systems, attitudes, and culturally embedded practices that cannot be adequately captured through biomedical or physiological measures [52]. This is highly relevant in CAM research, where health behaviours are strongly shaped by psychological factors, cultural norms, and subjective perceptions of treatment safety and efficacy. By employing validated instruments like the CAMBI [14] and MALMAS [15], this study ensured that constructs were measured reliably and in ways that have been proven applicable in real-world clinical and community settings [53]. Furthermore, adherence was operationalised dichotomously; this binary classification simplifies model training and clinical deployment, providing actionable outputs to guide interventions [54]. Previous studies successfully utilising similar surveys [55–57] collectively demonstrate that questionnaire-based methodologies provide both theoretical rigor in psychological measurement and practical feasibility for real-world clinical research applications, supporting the methodological framework employed in the current investigation.

Over the last 15 years, machine learning has been widely applied to predict medication non-adherence in chronic diseases such as diabetes [58], hypertension [57], and dyslipidaemia [59], with some frameworks successfully combining these conditions [60,61]. Systematic reviews confirm the broad application of diverse machine learning algorithms, including decision trees, boosting methods, and stacked ensembles, which consistently outperform conventional statistical methods by effectively handling complex, non-linear relationships in high dimensional data [57,62,63]. However, despite the maturity of machine learning in this field, existing adherence models have yet to incorporate CAM beliefs as predictors, nor have they utilised multiethnic, population specific datasets. This research marks a novel approach by introducing culturally grounded CAM constructs into an advanced machine learning predictive framework, addressing a distinct gap within the Malaysian and broader Asian context.

This study identified the stacked ensemble GLM trained on LR-selected variables with the unbalanced dataset as the best-performing model. The stacked ensemble approach inherently reduces model bias and variance [64], and the linear GLM meta-learner provided more stable, generalisable predictions by avoiding the overfitting risks associated with RF or GBM ensembles [60]. Maintaining the naturally unbalanced dataset proved superior to synthetic balancing via SMOTE-Tomek, which risks introducing noise and overfitting in small clinical datasets [65], while cross-validation ensured consistent performance across folds [66]. Furthermore, targeted feature selection improved predictive accuracy by reducing noise and multicollinearity [67]. LR-based selection was specifically superior to SVM and RF which suffer from tuning sensitivities and high-variance biases, respectively [68] because it produces interpretable, robust coefficients suitable for smaller datasets [61]. Ultimately, the synergy between LR selected variables and the GLM meta learner, both emphasising linear interpretability and stable weight assignment, optimised the model’s ability to synthesise diverse outputs, justifying its superiority and reliability in predicting medication non-adherence.

To interpret the model’s predictions and address the “black box” limitation of machine learning, SHAP analysis was applied [69]. This study’s findings confirmed conventional predictors identified in previous literature, such as educational level [31], regimen complexity [67], and treatment duration [70], but crucially extended the evidence to highlight the profound impact of psychosocial CAM constructs. Belief in natural treatments emerged as the strongest predictor of non-adherence. Patients who strongly believed natural remedies were more likely to substitute or prioritise these approaches over conventional medicine, reflecting cultural and religious perceptions in Asian societies that “natural” therapies are safer and spiritually aligned. This tendency often reduces their perceived necessity for conventional medicines, ultimately weakening adherence [71]. Belief in holistic health treatments followed a similar pattern. Patients with strong positive attitudes toward holistic, “whole-person” care were more likely to be non-adherent, emphasising how culturally embedded worldviews directly influence a patient’s willingness to continue prescribed regimens [72].

It is important to note that CAM users frequently engage in dual use, continuing to take prescribed conventional medications alongside alternative therapies [73]. However, despite this concurrent use, health psychology models such as the Necessity-Concerns Framework posit that strong trust in CAM efficacy systematically reduces a patient’s perceived necessity for conventional pharmacotherapy [9]. Empirical evidence across multiple chronic diseases confirms that patients with strong alternative beliefs, even if they are dual users, are significantly more likely to become non-adherent to their conventional regimens [74]. Therefore, a patient’s underlying belief system serves as a highly effective predictive marker for non-adherence risk, regardless of their physical co-use patterns. Consequently, the predictive framework established in this study can be utilised to proactively catch patients who are gradually abandoning their conventional therapy at an early stage, thereby enabling clinicians to deploy timely and targeted interventions before complete non-adherence occurs.

Sociodemographic and clinical variables also interacted intricately with these beliefs. Ethnicity played an important role, with indigenous patients demonstrating stronger adherence, likely reflecting trust in community-based health providers and the success of targeted public health efforts [75]. Educational level acted as a protective factor, as higher education facilitates better health literacy, counterbalancing misconceptions about modern medicine and reducing reliance on unverified CAM alternatives [50,76]. Clinically, higher regimen complexity (greater daily doses and number of medications) increased non-adherence risk due to the sheer burden of pill load and dosing frequency [77], a barrier often exacerbated for patients already leaning toward CAM. Conversely, longer treatment durations were associated with higher adherence, as these patients have had time to adapt to routines and experience clinical improvements that reinforce their trust in conventional therapy, whereas newer patients remain more vulnerable to side-effect concerns [78]. Finally, hypertension status showed a slight positive association with adherence, reflecting the long-standing integration of structured follow-ups and routine monitoring for this condition within Malaysian healthcare [79].

Taken together, these SHAP-based insights reveal how a combination of cultural beliefs (natural and holistic health perspectives, ethnicity, religion), social factors (education), and clinical variables (pill burden, treatment duration, disease status) interact to influence adherence. Importantly, they highlight novel pathways (particularly through CAM beliefs) that must be considered when designing adherence interventions for muti-ethnic Malaysian patients with chronic diseases.

Risk stratification plays a crucial role in predictive modelling, as it allows us to classify patients into higher and lower risk groups (in this study, non-adherent versus adherent) to guide appropriate intervention [80]. Misclassification in this context has different consequences depending on whether it is a false positive (FP) or a false negative (FN). FP, where adherent patients are predicted to be non-adherent, would result only in additional counselling, which is low-cost and low risk. FN, however, where non-adherent patients are misclassified as adherent, are more problematic because they lead to missed opportunities for intervention, greater medication wastage, and higher associated costs. Minimising false negatives (FN) is of paramount clinical importance to ensure at-risk patients are not overlooked. While the Youden Index identified a statistically optimal cutoff that balanced sensitivity and specificity, Decision Curve Analysis (DCA) suggested its maximum net benefit occurred at a substantially higher threshold. However, applying this higher cutoff would unacceptably increase FN, contradicting the study’s aim of improving adherence and reducing medication wastage. Because DCA evaluates net benefit across a continuum rather than prescribing a single definitive threshold [81], and the model maintained clear clinical utility above the “treat-all” strategy at the Youden-derived cutoff, selecting the lower threshold is justified. This approach represents an optimal compromise, aligning balanced diagnostic accuracy with practical clinical utility for effective patient risk stratification.

This study has several limitations. Medication adherence and CAM beliefs were self-reported, raising the risk of recall and social desirability bias. The cross-sectional design also limits causal interpretation, allowing only associations to be identified. Although the study sample captured a multi-ethnic Malaysian population with type 2 diabetes, hypertension, and dyslipidaemia, recruitment from a single healthcare setting restricts generalisability to rural, private, or broader Asia-Pacific populations. Furthermore, while SHAP analysis improved the interpretability of the ensemble model, unmeasured factors such as health literacy, family influence, and physician-patient communication were not assessed. Finally, although both balanced and unbalanced datasets were tested, the modest sample size in the validation cohort may limit robustness of subgroup predictions.

Future work should build on these findings using longitudinal designs to clarify causal pathways between CAM beliefs, treatment duration, and adherence. Broader recruitment across healthcare contexts, including rural and private settings, would strengthen external validity. Combining objective adherence measures (e.g., refill records, electronic pill monitoring) with self-report scales would also reduce measurement bias. Larger samples and external validation cohorts are needed to test the stability of ensemble methods, while additional predictors such as health literacy, cultural norms, and family decision-making roles could enrich future models. Importantly, as this study is the first to integrate CAM beliefs into machine learning adherence prediction, further research should explore specific CAM modalities and their interaction with ethnicity and religiosity to refine culturally tailored prediction and intervention strategies.

## Conclusion

This study demonstrates the novelty of integrating CAM beliefs and use into machine learning models for predicting medication non-adherence, addressing a critical issue that underlies the growing problem of medication wastage in chronic disease management. By applying advanced approaches such as stacked ensemble learning, this study not only achieved strong predictive performance but also provided interpretable insights into how cultural and belief-driven factors shape adherence. Importantly, the inclusion of religious and cultural influences particularly salient in multi-ethnic Asian contexts like Malaysia underscores that medication-taking behaviours cannot be disentangled from patients’ worldviews, where natural and holistic remedies are often perceived as safer and spiritually aligned. Using population-specific data from Malaysian patients with diabetes, hypertension, and dyslipidaemia, this work highlights the need for predictive models that reflect local cultural realities rather than relying on universal assumptions. By linking CAM beliefs, sociodemographic variables, and adherence patterns through machine learning, this study contributes a novel framework for understanding and mitigating medication wastage in culturally diverse populations.

Clinically, these findings highlight the importance of incorporating brief assessments of patient beliefs into routine clinical consultations, allowing healthcare providers to identify individuals at risk of non-adherence at an earlier stage. Instead of relying on a one-size-fits-all approach, clinicians and pharmacists can leverage these insights to develop culturally appropriate, patient-centred intervention strategies that acknowledge and respectfully address patients’ holistic health beliefs and treatment preferences. From a health policy perspective, the application of these predictive thresholds facilitates the more efficient and targeted deployment of counselling services and adherence-promoting interventions before non-adherence manifests. By proactively recognising patients who are highly likely to discontinue prescribed therapies in favour of alternative treatment options, healthcare systems can minimise the unnecessary dispensing of medications that may ultimately remain unused. This approach has the potential to substantially reduce both the economic costs and environmental consequences associated with medication wastage, while simultaneously enhancing medication adherence and improving long-term outcomes for individuals living with chronic diseases.

## Supporting information

S1 Table Hyperparameters of the models in the study. (DOCX)

S2 Table Overall results of the 120 models. (DOCX)

S1 File Extended methodology and model evaluation details. (DOCX)

S2 File De-identified data. (XLSX)

## References

[1] IDF Diabetes Atlas. 10th ed. Brussels: International Diabetes Federation. 2021.

[2] Institute for Public Health (IPH). National health and morbidity survey (NHMS) 2019: non-communicable diseases, healthcare demand, and health literacy. Kuala Lumpur: Ministry of Health Malaysia. 2019. https://www.moh.gov.my

[3] Sabaté E. Adherence to long-term therapies: evidence for action. Geneva: World Health Organization. 2003.

[4] Kleinsinger F. The Unmet Challenge of Medication Nonadherence. Perm J. 2018;22:18–033. doi: 10.7812/TPP/18-033 30005722 PMC6045499

[5] Farrukh MJ, Makmor-Bakry M, Hatah E, Jan TH. Impact of complementary and alternative medicines on antiepileptic medication adherence among epilepsy patients. BMC Complement Med Ther. 2021;21(1):50. doi: 10.1186/s12906-021-03224-2 33541336 PMC7863518

[6] Hasan SS, Ahmed SI, Bukhari NI, Loon WCW. Use of complementary and alternative medicine among patients with chronic diseases at outpatient clinics. Complement Ther Clin Pract. 2009;15(3):152–7. doi: 10.1016/j.ctcp.2009.02.003 19595416

[7] Che CT, George V, Ijinu TP, Pushpangadan P, Andrae-Marobela K. Traditional medicine. Pharmacognosy. Academic Press. 2024:11–28.

[8] Osterberg L, Blaschke T. Adherence to medication. N Engl J Med. 2005;353(5):487–97. 16079372 10.1056/NEJMra050100

[9] Ramdzan SN, Pinnock H, Liew SM, Sukri N, Salim H, Hanafi NS, et al. Perceptions of complementary/alternative medicine use and influence on evidence-based asthma medicine adherence in Malaysian children. NPJ Prim Care Respir Med. 2019;29(1):5. doi: 10.1038/s41533-019-0118-x 30804340 PMC6389881

[10] Kretchy IA, Owusu-Daaku FT, Danquah SA, Asampong E. A psychosocial perspective of medication side effects, experiences, coping approaches and implications for adherence in hypertension management. Clin Hypertens. 2015;21:19. doi: 10.1186/s40885-015-0028-3 26893929 PMC4750803

[11] Alfian S, Sukandar H, Arisanti N, Abdulah R. Complementary and alternative medicine use decreases adherence to prescribed medication in diabetes patients. Ann Trop Med Public Health. 2016;9(3):174. doi: 10.4103/1755-6783.179108

[12] Krousel-Wood MA, Muntner P, Joyce CJ, Islam T, Stanley E, Holt EW, et al. Adverse effects of complementary and alternative medicine on antihypertensive medication adherence: findings from the cohort study of medication adherence among older adults. J Am Geriatr Soc. 2010;58(1):54–61. doi: 10.1111/j.1532-5415.2009.02639.x 20122040 PMC2920063

[13] Chua SS, Lai PS, Tan CH, Chan SP, Chung WW, Morisky DE. The development and validation of the Malaysian medication adherence scale (MALMAS) among patients with 2 type diabetes in Malaysia. Int J Pharm Pharm Sci. 2013;5(3):790–4.

[14] Bishop FL, Yardley L, Lewith G. Developing a measure of treatment beliefs: the complementary and alternative medicine beliefs inventory. Complement Ther Med. 2005;13(2):144–9. doi: 10.1016/j.ctim.2005.01.005 16036173

[15] Ware JE Jr, Sherbourne CD. The MOS 36-item short-form health survey (SF-36). I. Conceptual framework and item selection. Med Care. 1992;30(6):473–83. 1593914

[16] Maimunah AH, Shamzaini S, Azura AA, Safura MM, Hidayati AN, Hadi MM, et al. A handbook of traditional and complementary medicine programme in Malaysia. Kuala Lumpur: Ministry of Health Malaysia. 2011.

[17] Chung WW, Chua SS, Lai PSM, Morisky DE. The Malaysian Medication Adherence Scale (MALMAS): Concurrent Validity Using a Clinical Measure among People with Type 2 Diabetes in Malaysia. PLoS One. 2015;10(4):e0124275. doi: 10.1371/journal.pone.0124275 25909363 PMC4409377

[18] Pargent F, Pfisterer F, Thomas J, Bischl B. Regularized target encoding outperforms traditional methods in supervised machine learning with high cardinality features. Comput Stat. 2022;37(5):2671–92. doi: 10.1007/s00180-022-01207-6

[19] Cateni S, Colla V. Variable selection for efficient design of machine learning-based models: efficient approaches for industrial applications. In: International Conference on Engineering Applications of Neural Networks, 2016. 352–66.

[20] Singh D, Singh B. Feature wise normalization: An effective way of normalizing data. Pattern Recognition. 2022;122:108307. doi: 10.1016/j.patcog.2021.108307

[21] Kuhn M, Johnson K. Applied predictive modeling. New York: Springer. 2013.

[22] Swana EF, Doorsamy W, Bokoro P. Tomek Link and SMOTE Approaches for Machine Fault Classification with an Imbalanced Dataset. Sensors (Basel). 2022;22(9):3246. doi: 10.3390/s22093246 35590937 PMC9099503

[23] Kushagra KR, Jain S. Feature selection for medical diagnosis using machine learning: A review. Computational intelligence for engineering and management applications: Select proceedings of CIEMA 2022. 2023:179–89.

[24] Rahman MS, Khan JA. Building a Robust Linear Model with Backward Elimination Procedure. Dhaka Univ J Sci. 2015;62(2):87–93. doi: 10.3329/dujs.v62i2.21971

[25] Cessie SL, Houwelingen JV. Ridge estimators in logistic regression. J R Stat Soc Ser C Appl Stat. 1992;41:191–201.

[26] Vapnik V, Guyon I, Hastie T. Support vector machines. Mach Learn. 1995;20:273–97.

[27] Cover T, Hart P. Nearest neighbor pattern classification. IEEE Trans Inform Theory. 1967;13(1):21–7. doi: 10.1109/tit.1967.1053964

[28] Zhu H, Williams CK, Rohwer R, Morciniec M. Gaussian regression and optimal finite dimensional linear models. 1997.

[29] Ontivero-Ortega M, Lage-Castellanos A, Valente G, Goebel R, Valdes-Sosa M. Fast Gaussian Naïve Bayes for searchlight classification analysis. Neuroimage. 2017;163:471–9. doi: 10.1016/j.neuroimage.2017.09.001 28877514

[30] McCallum A, Nigam K. A comparison of event models for naive bayes text classification. In: 1998. 41–8.

[31] Song YY, Lu Y. Decision tree methods: applications for classification and prediction. Shanghai Archives of Psychiatry. 2015. 10.11919/j.issn.1002-0829.215044 PMC4466856 26120265

[32] Breiman L. Classification and regression trees. Oxfordshire, England, UK: Routledge. 2017.

[33] Freund Y, Schapire RE. A Decision-Theoretic Generalization of On-Line Learning and an Application to Boosting. Journal of Computer and System Sciences. 1997;55(1):119–39. doi: 10.1006/jcss.1997.1504

[34] Friedman JH. Stochastic gradient boosting. Computational Statistics & Data Analysis. 2002;38(4):367–78. doi: 10.1016/s0167-9473(01)00065-2

[35] Breiman L. Bagging predictors. Mach Learn. 1996;24:123–40.

[36] Mohammed A, Kora R. A comprehensive review on ensemble deep learning: Opportunities and challenges. Journal of King Saud University - Computer and Information Sciences. 2023;35(2):757–74. doi: 10.1016/j.jksuci.2023.01.014

[37] Myers RH, Montgomery DC. A Tutorial on Generalized Linear Models. Journal of Quality Technology. 1997;29(3):274–91. doi: 10.1080/00224065.1997.11979769

[38] A G PV, K AK, Varadarajan V. Estimating Software Development Efforts Using a Random Forest-Based Stacked Ensemble Approach. Electronics. 2021;10(10):1195. doi: 10.3390/electronics10101195

[39] Natekin A, Knoll A. Gradient boosting machines, a tutorial. Front Neurorobot. 2013;7:21. doi: 10.3389/fnbot.2013.00021 24409142 PMC3885826

[40] Kanyongo W, Ezugwu AE. Feature selection and importance of predictors of non-communicable diseases medication adherence from machine learning research perspectives. Informatics in Medicine Unlocked. 2023;38:101232. doi: 10.1016/j.imu.2023.101232

[41] Mascha EJ. Identifying the Best Cut-Point for a Biomarker, or Not. Anesth Analg. 2018;127(4):820–2. doi: 10.1213/ANE.0000000000003680 30216286

[42] Vickers AJ, Elkin EB. Decision curve analysis: a novel method for evaluating prediction models. Med Decis Making. 2006;26(6):565–74. doi: 10.1177/0272989X06295361 17099194 PMC2577036

[43] Samawi HM, Yin J, Zhang X, Yu L, Rochani H, Vogel R, et al. Kullback-Leibler divergence for medical diagnostics accuracy and cut-point selection criterion: how it is related to the Youden index. J Appl Bioinform Comput Biol. 2020;9:2.

[44] Katki HA. Quantifying risk stratification provided by diagnostic tests and risk predictions: Comparison to AUC and decision curve analysis. Stat Med. 2019;38(16):2943–55. doi: 10.1002/sim.8163 31037749 PMC6827980

[45] Pablo CG, Austria KA, Cortez HN, Garcia KB, Julao KG, Pulido NA, et al. Medication adherence of hypertensive and diabetic. Journal of Social Health. 2018;1(1).

[46] Aktaş B, Berivan Bakan A. Relationship Between Attitudes about Medication Adherence and Complementary and Alternative Medicines in Elderly Individuals with Chronic Diseases. Altern Ther Health Med. 2021;27(4):14–8. 33421046

[47] Chantzaras A, Yfantopoulos J. Determinants of medication adherence in patients with diabetes, hypertension, and hyperlipidemia. Hormones (Athens). 2025;24(2):443–59. doi: 10.1007/s42000-025-00631-9 39971883 PMC12339649

[48] Almakadma AH, De Vol A, Alabdaljabar MS, Aldosari S, Muhsen I, AlFreihi O, et al. Complementary and alternative medicine use and its association with medication adherence in inflammatory bowel disease and other gastrointestinal diseases. Saudi Journal of Gastroenterology. 2023;29(4):233–9. doi: 10.4103/sjg.sjg_468_22 37282444 PMC10445501

[49] Cassidy S. Resilience building in students: The role of academic self-efficacy. Frontiers in Psychology. 2015;6:1781. 26640447 10.3389/fpsyg.2015.01781 PMC4661232

[50] Abdul Wahab NA, Makmor Bakry M, Ahmad M, Mohamad Noor Z, Mhd Ali A. Exploring Culture, Religiosity and Spirituality Influence on Antihypertensive Medication Adherence Among Specialised Population: A Qualitative Ethnographic Approach. Patient Prefer Adherence. 2021;15:2249–65. doi: 10.2147/PPA.S319469 34675490 PMC8502050

[51] Razali NH, Ali A, Hua Gan S, Sen Lim C. Prevalence of Traditional and Complementary Alternative Medicine’s Use among Cancer Patients in South Peninsular Malaysia. Asian Pac J Cancer Biol. 2020;5(1):19–26. doi: 10.31557/apjcb.2020.5.1.19-26

[52] Awad E, Ramji R, Cirovic S, Rämgård M, Kottorp A, Shleev S. Developing and evaluating non-invasive healthcare technologies for a group of female participants from a socioeconomically disadvantaged area. Sci Rep. 2021;11(1):23896. doi: 10.1038/s41598-021-03262-3 34903797 PMC8668900

[53] Intilangelo A, Majic S, Palchik V, Traverso ML. Validated medication adherence questionnaires and associated factors in chronic patients: systematic review. Farmacia Hospitalaria. 2024. 10.1016/j.farma.2024.04.019 38862302

[54] Walters B, Ortega-Martorell S, Olier I, Lisboa PJG. Towards interpretable machine learning for clinical decision support. In: 2022 International Joint Conference on Neural Networks (IJCNN), 2022. 1–8. 10.1109/ijcnn55064.2022.9892114

[55] Aziz F, Sooriamoorthy S, Liew B, Syed Ahmad SM, Chong WW, Malek S, et al. Preliminary study: Data analytics for predicting medication adherence in Malaysian arthritis patients. Digit Health. 2025;11. doi: 10.1177/20552076241309505 39996067 PMC11848903

[56] Aziz F, Malek S, Sooriamoorthy S, Mahamood IA, Wen CW, Syed Ahmad SM, et al. Predicting medication wastage using machine learning based on patient beliefs. Digit Health. 2025;11. doi: 10.1177/20552076251355127 40666624 PMC12260319

[57] Aziz F, Malek S, Ali AM, Wong MS, Mosleh M, Milow P. Determining hypertensive patients’ beliefs towards medication and associations with medication adherence using machine learning methods. PeerJ. 2020;8:e8286. doi: 10.7717/peerj.8286 PMC7075362 32206445

[58] Li M, Lu X, Yang H, Yuan R, Yang Y, Tong R, et al. Development and assessment of novel machine learning models to predict medication non-adherence risks in type 2 diabetics. Frontiers in Public Health. 2022;10:1000622. 36466490 10.3389/fpubh.2022.1000622 PMC9714465

[59] Lucas JE, Bazemore TC, Alo C, Monahan PB, Voora D. An electronic health record based model predicts statin adherence, LDL cholesterol, and cardiovascular disease in the United States Military Health System. PLoS One. 2017;12(11):e0187809. doi: 10.1371/journal.pone.0187809 29155848 PMC5695792

[60] Ploshchik I, Chatzimparmpas A, Kerren A. MetaStackVis: Visually-Assisted Performance Evaluation of Metamodels. In: 2023 IEEE 16th Pacific Visualization Symposium (PacificVis), 2023. 207–11. 10.1109/pacificvis56936.2023.00030

[61] Liu J, Wu X, Xie Y, Tang Z, Xie Y, Gong S. Small samples-oriented intrinsically explainable machine learning using Variational Bayesian Logistic Regression: An intensive care unit readmission prediction case for liver transplantation patients. Expert Systems with Applications. 2024;235:121138. doi: 10.1016/j.eswa.2023.121138

[62] Mohammed Hassoon I. Boosting Learning Algorithms for Chronic Diseases Prediction: A Review. IJCI. 2024;50(2):22–30. doi: 10.25195/ijci.v50i2.506

[63] Zoakah N, Shey Nsang A, Ajibesin A, Zoakah A. Comparative Performance Analysis of Selected Machine Learning Algorithms and the Stacking Ensemble Method for Prediction of the Type II Diabetes Disease. Gazi University Journal of Science Part A: Engineering and Innovation. 2024;11(3):622–46. doi: 10.54287/gujsa.1531997

[64] Han SW, Hwang SY, Lee S. Ensemble approach for improving prediction in kernel regression and classification. Communications for Statistical Applications and Methods. 2016. doi: 10.5351/CSAM.2016.23.4.355

[65] Li M, Zhou H, Liu Q, Shao Y, Wang G. Informed Nonlinear Granular Ball Oversampling Framework for Noisy Imbalanced Classification. arXiv preprint. 2023. doi: 10.48550/arXiv.2307.01224

[66] Xu N, Hong J, Fisher TCG. Stability of cross-validation and minmax-optimal number of folds. 2017. https://arxiv.org/abs/1705.07349

[67] Park J, Kim Y. The Effect of Hypertension, Diabetes, and Hyperlipidemia on Medication Intake and Adherence: Analysis from Korean Health Panel Survey 2014-2017. Iran J Public Health. 2023;52(2):340–9. doi: 10.18502/ijph.v52i2.11887 37089158 PMC10113574

[68] Sanchez-Pinto LN, Venable LR, Fahrenbach J, Churpek MM. Comparison of variable selection methods for clinical predictive modeling. Int J Med Inform. 2018;116:10–7. doi: 10.1016/j.ijmedinf.2018.05.006 29887230 PMC6003624

[69] Sixian L, Imamura Y, Ahmed A. Application of Shapley Additive Explanation towards Determining Personalized Triage from Health Checkup Data. In: International Conference on Pervasive Computing Technologies for Healthcare, 2022. 496–509. Cham: Springer Nature Switzerland.

[70] Koohestani HR, Baghcheghi N. The Relationship Between the Use of Medicinal Plants and Medication Adherence in the Elderly with Chronic Diseases. Iranian Journal of Ageing. 2022;17(2):276–89.

[71] Kong Y, Shaver LG, Shi F, Yang L, Zhang W, Wei X, et al. Attitudes of Chinese immigrants in Canada towards the use of Traditional Chinese Medicine for prevention and management of COVID-19: a cross-sectional survey during the early stages of the pandemic. BMJ Open. 2021;11(9):e051499. doi: 10.1136/bmjopen-2021-051499 34521675 PMC8441218

[72] Purvis S, Manias E, Redley B. A Mixed-Methods Study of the Experiences and Beliefs of Older People With Complex Health Care Needs About Medication Adherence. J Nurs Care Qual. 2021;36(4):369–75. doi: 10.1097/NCQ.0000000000000522 33079818

[73] Martinez Sanchez LM, Martínez Domínguez GI, Molina Valencia JL, Vallejo Agudelo EO, Gallego González D, Pérez Palacio MI, et al. Uso de terapias alternativas y complementarias en pacientes con dolor crónico en una institución hospitalaria, Medellín, Colombia, 2014. Rev Soc Esp Dolor. 2016;23(6). doi: 10.20986/resed.2016.3451/2016

[74] Farrukh MJ, Makmor-Bakry M, Hatah E, Jan TH. Impact of complementary and alternative medicines on antiepileptic medication adherence among epilepsy patients. BMC Complement Med Ther. 2021;21(1):50. doi: 10.1186/s12906-021-03224-2 33541336 PMC7863518

[75] Johny AK, Cheah WL, Razitasham S. Disclosure of Traditional and Complementary Medicine Use and Its Associated Factors to Medical Doctor in Primary Care Clinics in Kuching Division, Sarawak, Malaysia. Evid Based Complement Alternat Med. 2017;2017:5146478. doi: 10.1155/2017/5146478 28529529 PMC5424170

[76] Zakaria AF, Sharoni SK, Fauzi R, Liang B. The usage of complementary and alternative medicine (CAM): prevalence, health literacy, beliefs and self-management among people with hypertension in a rural area, Pahang. Journal of Health and Translational Medicine. 2023;2023(September 15):86–95.

[77] del Rio C. Art pill burden and dosing frequency: do they matter? NEJM Journal Watch. 2014.

[78] Kassaw AT, Minyihun A, Gebreslassie BM. Medication regimen complexity and medication adherence among patients with multimorbidity treated at University of Gondar Compressive Specialized Hospital. Journal of Multimorbidity and Comorbidity. 2023.

[79] Leong MD, Ismail TS, Hwa GT. Anti-hypertensive prescription practices in private hospitals in Malaysia: a prospective, non-interventional, observational study. MJM. 2023;78(3):350. 37271845

[80] Luo G, Stone BL, Sakaguchi F, Sheng X, Murtaugh MA. Using Computational Approaches to Improve Risk-Stratified Patient Management: Rationale and Methods. JMIR Res Protoc. 2015;4(4):e128. doi: 10.2196/resprot.5039 26503357 PMC4704915

[81] Zhang Z, Rousson V, Lee W-C, Ferdynus C, Chen M, Qian X, et al. Decision curve analysis: a technical note. Ann Transl Med. 2018;6(15):308. doi: 10.21037/atm.2018.07.02 30211196 PMC6123195

