# Supplementary material for: Predicting medication non-adherence using machine learning: Incorporating Complementary and Alternative Medicine (CAM) beliefs in Malaysian chronic disease patients
Source: PLoS One. 2026 Jul 30;21(7):e0354682. doi: 10.1371/journal.pone.0354682 (PMC13423157; doi:10.1371/journal.pone.0354682)
Supplement: S1 Table — (DOCX) [file pone.0354682.s001.docx]

**S1 Table.** Hyperparameters of the models in the study.

| **Model** | **Hyperparameter** | **Values** |
| --- | --- | --- |
| **Logistic Regression** | Cs | {1, 10, 100} |
|  | penalty | {l2} |
|  | solver | {lbfgs, liblinear} |
| **AdaBoost** | n_estimators | {50, 100, 200} |
|  | learning_rate | {0.01, 0.1, 1.0} |
| **Bagging** | n_estimators | {10, 50, 100} |
|  | max_samples | {0.5, 0.7, 1.0} |
|  | max_features | {0.5, 0.7, 1.0} |
| **Gradient Boosting** | n_estimators | {100, 200} |
|  | learning_rate | {0.01, 0.1, 0.2} |
|  | max_depth | {3, 5, 7} |
|  | subsample | {0.7, 1.0} |
| **Random Forest** | n_estimators | {100, 200, 500} |
|  | max_depth | {5, 10, None} |
|  | min_samples_split | {2, 5, 10} |
|  | min_samples_leaf | {1, 2, 4} |
|  | bootstrap | {True, False} |
| **Gaussian Process** | kernel | {1×RBF(), 1×DotProduct(), 1×Matern(), 1×RationalQuadratic(), 1×WhiteKernel()} |
| **SVM (Linear)** | C | {0.1, 1, 10} |
| **SVM (Radial)** | C | {0.1, 1, 10} |
|  | gamma | {scale, auto} |
| **Decision Tree** | max_depth | {None, 10, 20, 30} |
|  | min_samples_split | {2, 10, 20} |
|  | min_samples_leaf | {1, 2, 4} |
| **Bernoulli Naïve Bayes** | alpha | {0.1, 1.0, 10.0} |
|  | binarize | {0.0, 0.5} |
| **Gaussian Naïve Bayes** | var_smoothing | {1e-9, 1e-8, 1e-7} |
| **K-Nearest Neighbors** | n_neighbors | {3, 5, 7} |
|  | weights | {uniform, distance} |
|  | metric | {euclidean, manhattan} |
| **Ensemble GLM** | final_estimator__C | {0.1, 1, 10, 100} |
|  | final_estimator__penalty | {l1, l2} |
|  | final_estimator__solver | {liblinear, saga} |
| **Ensemble Random Forest** | final_estimator__n_estimators | {100, 200, 500} |
|  | final_estimator__max_depth | {5, 10, None} |
|  | final_estimator__min_samples_split | {2, 5, 10} |
|  | final_estimator__min_samples_leaf | {1, 2, 4} |
|  | final_estimator__bootstrap | {True, False} |
| **Ensemble Gradient Boosting** | final_estimator__n_estimators | {100, 200} |
|  | final_estimator__learning_rate | {0.01, 0.1, 0.2} |
|  | final_estimator__max_depth | {3, 5, 7} |
|  | final_estimator__subsample | {0.7, 1.0} |
