# Supplementary material for: Predicting medication non-adherence using machine learning: Incorporating Complementary and Alternative Medicine (CAM) beliefs in Malaysian chronic disease patients
Source: PLoS One. 2026 Jul 30;21(7):e0354682. doi: 10.1371/journal.pone.0354682 (PMC13423157; doi:10.1371/journal.pone.0354682)
Supplement: S2 Table — (DOCX) [file pone.0354682.s002.docx]

**S2 Table.** Overall results of the 120 models.

**All Variables (Balanced Dataset)**

| Models | Training CV AUC | Testing CV AUC | Testing Accuracy | Testing Sensitivity | Testing Specificity | Testing PPV | Testing NPV | McNemar P-Value | Balanced Accuracy | PR-AUC |
| --- | --- | --- | --- | --- | --- | --- | --- | --- | --- | --- |
| Gaussian NB | 0.849 | 0.791 (0.702 - 0.879) | 0.765 (0.673 - 0.858) | 0.848 | 0.657 | 0.767 | 0.765 | 0.359 | 0.752 | 0.78 |
| Ada Boost | 0.854 | 0.788 (0.699 - 0.877) | 0.728 (0.632 - 0.825) | 0.739 | 0.714 | 0.676 | 0.773 | 0.832 | 0.727 | 0.748 |
| Bernoulli NB | 0.733 | 0.783 (0.693 - 0.872) | 0.728 (0.632 - 0.825) | 0.761 | 0.686 | 0.686 | 0.761 | 1 | 0.723 | 0.77 |
| SVM (Radial) | 0.861 | 0.777 (0.686 - 0.868) | 0.728 (0.632 - 0.825) | 0.783 | 0.657 | 0.697 | 0.75 | 0.832 | 0.72 | 0.68 |
| Gaussian Process | 0.806 | 0.774 (0.683 - 0.865) | 0.728 (0.632 - 0.825) | 0.783 | 0.657 | 0.697 | 0.75 | 0.832 | 0.72 | 0.706 |
| SVM (Linear) | 0.863 | 0.772 (0.681 - 0.863) | 0.716 (0.618 - 0.814) | 0.739 | 0.686 | 0.667 | 0.756 | 1 | 0.712 | 0.718 |
| Ensemble GLM | 0.878 | 0.768 (0.676 - 0.860) | 0.716 (0.618 - 0.814) | 0.717 | 0.714 | 0.658 | 0.767 | 0.678 | 0.716 | 0.732 |
| Random Forest | 0.891 | 0.768 (0.676 - 0.860) | 0.704 (0.604 - 0.803) | 0.739 | 0.657 | 0.657 | 0.739 | 1 | 0.698 | 0.724 |
| Logistic Regression | 0.845 | 0.766 (0.674 - 0.858) | 0.716 (0.618 - 0.814) | 0.739 | 0.686 | 0.667 | 0.756 | 1 | 0.712 | 0.72 |
| Ensemble RF | 0.874 | 0.765 (0.672 - 0.857) | 0.728 (0.632 - 0.825) | 0.783 | 0.657 | 0.697 | 0.75 | 0.832 | 0.72 | 0.721 |
| Bagging | 0.882 | 0.764 (0.671 - 0.856) | 0.704 (0.604 - 0.803) | 0.761 | 0.629 | 0.667 | 0.729 | 0.839 | 0.695 | 0.717 |
| Ensemble GBM | 0.856 | 0.743 (0.648 - 0.838) | 0.691 (0.591 - 0.792) | 0.761 | 0.6 | 0.656 | 0.714 | 0.69 | 0.68 | 0.717 |
| KNN | 0.842 | 0.741 (0.646 - 0.836) | 0.679 (0.577 - 0.781) | 0.63 | 0.743 | 0.605 | 0.763 | 0.169 | 0.687 | 0.668 |
| Decision Tree | 0.825 | 0.741 (0.645 - 0.836) | 0.741 (0.645 - 0.836) | 0.804 | 0.657 | 0.719 | 0.755 | 0.664 | 0.731 | 0.761 |
| Gradient Boosting | 0.884 | 0.717 (0.619 - 0.815) | 0.630 (0.524 - 0.735) | 0.717 | 0.514 | 0.581 | 0.66 | 0.585 | 0.616 | 0.663 |

**SVM-Selected Variables (Balanced Dataset)**

| Models | Training CV AUC | Testing CV AUC | Testing Accuracy | Testing Sensitivity | Testing Specificity | Testing PPV | Testing NPV | McNemar P-Value | Balanced Accuracy | PR-AUC |
| --- | --- | --- | --- | --- | --- | --- | --- | --- | --- | --- |
| KNN | 0.807 | 0.788 (0.699 - 0.877) | 0.704 (0.604 - 0.803) | 0.783 | 0.6 | 0.677 | 0.72 | 0.541 | 0.691 | 0.763 |
| Logistic Regression | 0.839 | 0.777 (0.687 - 0.868) | 0.728 (0.632 - 0.825) | 0.761 | 0.686 | 0.686 | 0.761 | 1 | 0.723 | 0.729 |
| Gaussian Process | 0.822 | 0.775 (0.685 - 0.866) | 0.716 (0.618 - 0.814) | 0.739 | 0.686 | 0.667 | 0.756 | 1 | 0.712 | 0.72 |
| Ada Boost | 0.864 | 0.768 (0.676 - 0.860) | 0.691 (0.591 - 0.792) | 0.696 | 0.686 | 0.632 | 0.744 | 0.69 | 0.691 | 0.706 |
| SVM (Linear) | 0.882 | 0.762 (0.670 - 0.855) | 0.716 (0.618 - 0.814) | 0.696 | 0.743 | 0.65 | 0.78 | 0.405 | 0.719 | 0.71 |
| Ensemble RF | 0.904 | 0.762 (0.669 - 0.855) | 0.728 (0.632 - 0.825) | 0.717 | 0.743 | 0.667 | 0.786 | 0.523 | 0.73 | 0.738 |
| Gaussian NB | 0.845 | 0.759 (0.666 - 0.852) | 0.753 (0.659 - 0.847) | 0.804 | 0.686 | 0.727 | 0.771 | 0.824 | 0.745 | 0.731 |
| Bernoulli NB | 0.716 | 0.752 (0.658 - 0.846) | 0.667 (0.564 - 0.769) | 0.63 | 0.714 | 0.595 | 0.744 | 0.248 | 0.672 | 0.743 |
| Decision Tree | 0.803 | 0.750 (0.656 - 0.844) | 0.691 (0.591 - 0.792) | 0.717 | 0.657 | 0.639 | 0.733 | 1 | 0.687 | 0.72 |
| SVM (Radial) | 0.825 | 0.745 (0.650 - 0.840) | 0.704 (0.604 - 0.803) | 0.696 | 0.714 | 0.641 | 0.762 | 0.541 | 0.705 | 0.635 |
| Ensemble GLM | 0.892 | 0.737 (0.641 - 0.833) | 0.679 (0.577 - 0.781) | 0.652 | 0.714 | 0.61 | 0.75 | 0.327 | 0.683 | 0.661 |
| Bagging | 0.848 | 0.712 (0.614 - 0.811) | 0.642 (0.538 - 0.746) | 0.609 | 0.686 | 0.571 | 0.718 | 0.265 | 0.647 | 0.627 |
| Random Forest | 0.897 | 0.707 (0.608 - 0.807) | 0.630 (0.524 - 0.735) | 0.609 | 0.657 | 0.561 | 0.7 | 0.362 | 0.633 | 0.644 |
| Ensemble GBM | 0.879 | 0.706 (0.607 - 0.805) | 0.691 (0.591 - 0.792) | 0.696 | 0.686 | 0.632 | 0.744 | 0.69 | 0.691 | 0.654 |
| Gradient Boosting | 0.855 | 0.672 (0.570 - 0.774) | 0.654 (0.551 - 0.758) | 0.652 | 0.657 | 0.59 | 0.714 | 0.572 | 0.655 | 0.593 |

**LR-Selected Variables (Balanced Dataset)**

| Models | Training CV AUC | Testing CV AUC | Testing Accuracy | Testing Sensitivity | Testing Specificity | Testing PPV | Testing NPV | McNemar P-Value | Balanced Accuracy | PR-AUC |
| --- | --- | --- | --- | --- | --- | --- | --- | --- | --- | --- |
| Ensemble RF | 0.903 | 0.781 (0.691 - 0.871) | 0.716 (0.618 - 0.814) | 0.761 | 0.657 | 0.676 | 0.745 | 1 | 0.709 | 0.777 |
| Ensemble GLM | 0.885 | 0.773 (0.681 - 0.864) | 0.679 (0.577 - 0.781) | 0.717 | 0.629 | 0.629 | 0.717 | 1 | 0.673 | 0.757 |
| SVM (Linear) | 0.884 | 0.772 (0.681 - 0.863) | 0.728 (0.632 - 0.825) | 0.761 | 0.686 | 0.686 | 0.761 | 1 | 0.723 | 0.746 |
| Ensemble GBM | 0.879 | 0.772 (0.681 - 0.863) | 0.704 (0.604 - 0.803) | 0.783 | 0.6 | 0.677 | 0.72 | 0.541 | 0.691 | 0.779 |
| KNN | 0.842 | 0.771 (0.680 - 0.863) | 0.728 (0.632 - 0.825) | 0.783 | 0.657 | 0.697 | 0.75 | 0.832 | 0.72 | 0.729 |
| Gaussian NB | 0.871 | 0.768 (0.676 - 0.860) | 0.728 (0.632 - 0.825) | 0.783 | 0.657 | 0.697 | 0.75 | 0.832 | 0.72 | 0.767 |
| Logistic Regression | 0.841 | 0.765 (0.673 - 0.858) | 0.716 (0.618 - 0.814) | 0.761 | 0.657 | 0.676 | 0.745 | 1 | 0.709 | 0.738 |
| Gaussian Process | 0.818 | 0.765 (0.672 - 0.857) | 0.716 (0.618 - 0.814) | 0.761 | 0.657 | 0.676 | 0.745 | 1 | 0.709 | 0.74 |
| Bernoulli NB | 0.739 | 0.753 (0.659 - 0.847) | 0.691 (0.591 - 0.792) | 0.761 | 0.6 | 0.656 | 0.714 | 0.69 | 0.68 | 0.743 |
| Random Forest | 0.892 | 0.747 (0.652 - 0.841) | 0.728 (0.632 - 0.825) | 0.739 | 0.714 | 0.676 | 0.773 | 0.832 | 0.727 | 0.707 |
| Bagging | 0.888 | 0.742 (0.647 - 0.837) | 0.667 (0.564 - 0.769) | 0.696 | 0.629 | 0.611 | 0.711 | 1 | 0.662 | 0.697 |
| SVM (Radial) | 0.839 | 0.732 (0.636 - 0.829) | 0.716 (0.618 - 0.814) | 0.739 | 0.686 | 0.667 | 0.756 | 1 | 0.712 | 0.616 |
| Ada Boost | 0.857 | 0.729 (0.632 - 0.825) | 0.716 (0.618 - 0.814) | 0.717 | 0.714 | 0.658 | 0.767 | 0.678 | 0.716 | 0.714 |
| Decision Tree | 0.822 | 0.707 (0.608 - 0.806) | 0.679 (0.577 - 0.781) | 0.717 | 0.629 | 0.629 | 0.717 | 1 | 0.673 | 0.715 |
| Gradient Boosting | 0.877 | 0.674 (0.572 - 0.776) | 0.642 (0.538 - 0.746) | 0.696 | 0.571 | 0.588 | 0.681 | 1 | 0.634 | 0.624 |

**RF-Selected Variables (Balanced Dataset)**

| Models | Training CV AUC | Testing CV AUC | Testing Accuracy | Testing Sensitivity | Testing Specificity | Testing PPV | Testing NPV | McNemar P-Value | Balanced Accuracy | PR-AUC |
| --- | --- | --- | --- | --- | --- | --- | --- | --- | --- | --- |
| Gaussian NB | 0.882 | 0.775 (0.684 - 0.866) | 0.728 (0.632 - 0.825) | 0.783 | 0.657 | 0.697 | 0.75 | 0.832 | 0.72 | 0.748 |
| Random Forest | 0.907 | 0.770 (0.679 - 0.862) | 0.704 (0.604 - 0.803) | 0.717 | 0.686 | 0.649 | 0.75 | 0.839 | 0.702 | 0.734 |
| Ensemble GLM | 0.902 | 0.757 (0.664 - 0.851) | 0.704 (0.604 - 0.803) | 0.761 | 0.629 | 0.667 | 0.729 | 0.839 | 0.695 | 0.719 |
| Gaussian Process | 0.814 | 0.751 (0.657 - 0.845) | 0.679 (0.577 - 0.781) | 0.652 | 0.714 | 0.61 | 0.75 | 0.327 | 0.683 | 0.684 |
| Ensemble RF | 0.895 | 0.749 (0.654 - 0.843) | 0.691 (0.591 - 0.792) | 0.696 | 0.686 | 0.632 | 0.744 | 0.69 | 0.691 | 0.696 |
| Ensemble GBM | 0.88 | 0.746 (0.651 - 0.840) | 0.679 (0.577 - 0.781) | 0.696 | 0.657 | 0.622 | 0.727 | 0.845 | 0.676 | 0.705 |
| Bagging | 0.888 | 0.743 (0.648 - 0.838) | 0.716 (0.618 - 0.814) | 0.717 | 0.714 | 0.658 | 0.767 | 0.678 | 0.716 | 0.698 |
| KNN | 0.879 | 0.737 (0.641 - 0.833) | 0.667 (0.564 - 0.769) | 0.63 | 0.714 | 0.595 | 0.744 | 0.248 | 0.672 | 0.705 |
| Decision Tree | 0.816 | 0.734 (0.637 - 0.830) | 0.716 (0.618 - 0.814) | 0.783 | 0.629 | 0.688 | 0.735 | 0.678 | 0.706 | 0.75 |
| Bernoulli NB | 0.738 | 0.727 (0.630 - 0.824) | 0.654 (0.551 - 0.758) | 0.63 | 0.686 | 0.585 | 0.725 | 0.345 | 0.658 | 0.716 |
| Logistic Regression | 0.869 | 0.726 (0.629 - 0.823) | 0.704 (0.604 - 0.803) | 0.739 | 0.657 | 0.657 | 0.739 | 1 | 0.698 | 0.61 |
| SVM (Radial) | 0.875 | 0.719 (0.621 - 0.817) | 0.691 (0.591 - 0.792) | 0.674 | 0.714 | 0.625 | 0.756 | 0.424 | 0.694 | 0.596 |
| SVM (Linear) | 0.873 | 0.715 (0.617 - 0.813) | 0.691 (0.591 - 0.792) | 0.717 | 0.657 | 0.639 | 0.733 | 1 | 0.687 | 0.598 |
| Gradient Boosting | 0.893 | 0.712 (0.614 - 0.811) | 0.679 (0.577 - 0.781) | 0.717 | 0.629 | 0.629 | 0.717 | 1 | 0.673 | 0.667 |
| Ada Boost | 0.858 | 0.702 (0.602 - 0.801) | 0.704 (0.604 - 0.803) | 0.739 | 0.657 | 0.657 | 0.739 | 1 | 0.698 | 0.665 |

**All Variables (Unbalanced Dataset)**

| Models | Training CV AUC | Testing CV AUC | Testing Accuracy | Testing Sensitivity | Testing Specificity | Testing PPV | Testing NPV | McNemar P-Value | Balanced Accuracy | PR-AUC |
| --- | --- | --- | --- | --- | --- | --- | --- | --- | --- | --- |
| Gaussian NB | 0.831 | 0.812 (0.727 - 0.897) | 0.765 (0.673 - 0.858) | 0.87 | 0.629 | 0.786 | 0.755 | 0.167 | 0.749 | 0.803 |
| SVM (Linear) | 0.828 | 0.811 (0.725 - 0.896) | 0.741 (0.645 - 0.836) | 0.804 | 0.657 | 0.719 | 0.755 | 0.664 | 0.731 | 0.796 |
| Gaussian Process | 0.772 | 0.809 (0.724 - 0.895) | 0.765 (0.673 - 0.858) | 0.826 | 0.686 | 0.75 | 0.776 | 0.648 | 0.756 | 0.786 |
| Ensemble GBM | 0.789 | 0.807 (0.722 - 0.893) | 0.765 (0.673 - 0.858) | 0.804 | 0.714 | 0.735 | 0.787 | 1 | 0.759 | 0.768 |
| Ensemble GLM | 0.805 | 0.807 (0.722 - 0.893) | 0.753 (0.659 - 0.847) | 0.848 | 0.629 | 0.759 | 0.75 | 0.263 | 0.738 | 0.746 |
| Logistic Regression | 0.773 | 0.806 (0.720 - 0.892) | 0.728 (0.632 - 0.825) | 0.804 | 0.629 | 0.71 | 0.74 | 0.523 | 0.716 | 0.785 |
| SVM (Radial) | 0.825 | 0.806 (0.719 - 0.892) | 0.741 (0.645 - 0.836) | 0.804 | 0.657 | 0.719 | 0.755 | 0.664 | 0.731 | 0.773 |
| Random Forest | 0.829 | 0.803 (0.717 - 0.890) | 0.765 (0.673 - 0.858) | 0.804 | 0.714 | 0.735 | 0.787 | 1 | 0.759 | 0.784 |
| Bernoulli NB | 0.703 | 0.799 (0.711 - 0.886) | 0.741 (0.645 - 0.836) | 0.804 | 0.657 | 0.719 | 0.755 | 0.664 | 0.731 | 0.791 |
| Ensemble RF | 0.796 | 0.798 (0.711 - 0.886) | 0.765 (0.673 - 0.858) | 0.826 | 0.686 | 0.75 | 0.776 | 0.648 | 0.756 | 0.772 |
| Ada Boost | 0.828 | 0.797 (0.709 - 0.885) | 0.765 (0.673 - 0.858) | 0.804 | 0.714 | 0.735 | 0.787 | 1 | 0.759 | 0.764 |
| Bagging | 0.805 | 0.788 (0.699 - 0.877) | 0.778 (0.687 - 0.868) | 0.826 | 0.714 | 0.758 | 0.792 | 0.815 | 0.77 | 0.755 |
| Gradient Boosting | 0.791 | 0.774 (0.683 - 0.865) | 0.741 (0.645 - 0.836) | 0.826 | 0.629 | 0.733 | 0.745 | 0.383 | 0.727 | 0.737 |
| KNN | 0.785 | 0.741 (0.646 - 0.836) | 0.716 (0.618 - 0.814) | 0.739 | 0.686 | 0.667 | 0.756 | 1 | 0.712 | 0.632 |
| Decision Tree | 0.702 | 0.740 (0.645 - 0.836) | 0.642 (0.538 - 0.746) | 0.761 | 0.486 | 0.607 | 0.66 | 0.265 | 0.623 | 0.702 |

**SVM-Selected Variables (Unbalanced Dataset)**

| Models | Training CV AUC | Testing CV AUC | Testing Accuracy | Testing Sensitivity | Testing Specificity | Testing PPV | Testing NPV | McNemar P-Value | Balanced Accuracy | PR-AUC |
| --- | --- | --- | --- | --- | --- | --- | --- | --- | --- | --- |
| Ensemble GLM | 0.808 | 0.816 (0.732 - 0.901) | 0.802 (0.716 - 0.889) | 0.848 | 0.743 | 0.788 | 0.812 | 0.804 | 0.795 | 0.781 |
| SVM (Linear) | 0.835 | 0.808 (0.723 - 0.894) | 0.802 (0.716 - 0.889) | 0.848 | 0.743 | 0.788 | 0.812 | 0.804 | 0.795 | 0.772 |
| Logistic Regression | 0.778 | 0.798 (0.710 - 0.885) | 0.753 (0.659 - 0.847) | 0.804 | 0.686 | 0.727 | 0.771 | 0.824 | 0.745 | 0.749 |
| Gaussian Process | 0.765 | 0.793 (0.705 - 0.881) | 0.728 (0.632 - 0.825) | 0.739 | 0.714 | 0.676 | 0.773 | 0.832 | 0.727 | 0.743 |
| Gaussian NB | 0.809 | 0.788 (0.699 - 0.877) | 0.765 (0.673 - 0.858) | 0.826 | 0.686 | 0.75 | 0.776 | 0.648 | 0.756 | 0.756 |
| Ada Boost | 0.81 | 0.786 (0.697 - 0.876) | 0.753 (0.659 - 0.847) | 0.783 | 0.714 | 0.714 | 0.783 | 1 | 0.748 | 0.755 |
| SVM (Radial) | 0.789 | 0.786 (0.696 - 0.875) | 0.728 (0.632 - 0.825) | 0.804 | 0.629 | 0.71 | 0.74 | 0.523 | 0.716 | 0.749 |
| KNN | 0.751 | 0.768 (0.676 - 0.860) | 0.728 (0.632 - 0.825) | 0.826 | 0.6 | 0.724 | 0.731 | 0.286 | 0.713 | 0.769 |
| Gradient Boosting | 0.775 | 0.758 (0.665 - 0.852) | 0.716 (0.618 - 0.814) | 0.739 | 0.686 | 0.667 | 0.756 | 1 | 0.712 | 0.679 |
| Bernoulli NB | 0.689 | 0.752 (0.658 - 0.846) | 0.667 (0.564 - 0.769) | 0.63 | 0.714 | 0.595 | 0.744 | 0.248 | 0.672 | 0.743 |
| Ensemble RF | 0.82 | 0.750 (0.656 - 0.844) | 0.704 (0.604 - 0.803) | 0.696 | 0.714 | 0.641 | 0.762 | 0.541 | 0.705 | 0.668 |
| Random Forest | 0.825 | 0.742 (0.646 - 0.837) | 0.679 (0.577 - 0.781) | 0.696 | 0.657 | 0.622 | 0.727 | 0.845 | 0.676 | 0.651 |
| Ensemble GBM | 0.79 | 0.723 (0.626 - 0.821) | 0.691 (0.591 - 0.792) | 0.696 | 0.686 | 0.632 | 0.744 | 0.69 | 0.691 | 0.659 |
| Bagging | 0.79 | 0.707 (0.608 - 0.806) | 0.679 (0.577 - 0.781) | 0.739 | 0.6 | 0.636 | 0.708 | 0.845 | 0.67 | 0.642 |
| Decision Tree | 0.763 | 0.651 (0.547 - 0.755) | 0.667 (0.564 - 0.769) | 0.739 | 0.571 | 0.625 | 0.694 | 0.701 | 0.655 | 0.644 |

**LR-Selected Variables (Unbalanced Dataset)**

| Models | Training CV AUC | Testing CV AUC | Testing Accuracy | Testing Sensitivity | Testing Specificity | Testing PPV | Testing NPV | McNemar P-Value | Balanced Accuracy | PR-AUC |
| --- | --- | --- | --- | --- | --- | --- | --- | --- | --- | --- |
| Ensemble GLM | 0.803 | 0.816 (0.731 - 0.900) | 0.778 (0.687 - 0.868) | 0.848 | 0.686 | 0.774 | 0.78 | 0.481 | 0.767 | 0.785 |
| SVM (Linear) | 0.824 | 0.799 (0.711 - 0.886) | 0.778 (0.687 - 0.868) | 0.848 | 0.686 | 0.774 | 0.78 | 0.481 | 0.767 | 0.766 |
| Logistic Regression | 0.774 | 0.794 (0.706 - 0.882) | 0.765 (0.673 - 0.858) | 0.848 | 0.657 | 0.767 | 0.765 | 0.359 | 0.752 | 0.762 |
| Ada Boost | 0.8 | 0.790 (0.702 - 0.879) | 0.741 (0.645 - 0.836) | 0.783 | 0.686 | 0.706 | 0.766 | 1 | 0.734 | 0.738 |
| Gaussian NB | 0.812 | 0.787 (0.698 - 0.876) | 0.741 (0.645 - 0.836) | 0.848 | 0.6 | 0.75 | 0.736 | 0.189 | 0.724 | 0.782 |
| SVM (Radial) | 0.799 | 0.786 (0.697 - 0.876) | 0.741 (0.645 - 0.836) | 0.804 | 0.657 | 0.719 | 0.755 | 0.664 | 0.731 | 0.759 |
| Gaussian Process | 0.761 | 0.783 (0.693 - 0.872) | 0.753 (0.659 - 0.847) | 0.804 | 0.686 | 0.727 | 0.771 | 0.824 | 0.745 | 0.745 |
| KNN | 0.771 | 0.767 (0.675 - 0.859) | 0.716 (0.618 - 0.814) | 0.804 | 0.6 | 0.7 | 0.725 | 0.405 | 0.702 | 0.743 |
| Random Forest | 0.803 | 0.766 (0.674 - 0.859) | 0.716 (0.618 - 0.814) | 0.804 | 0.6 | 0.7 | 0.725 | 0.405 | 0.702 | 0.737 |
| Ensemble GBM | 0.777 | 0.761 (0.669 - 0.854) | 0.704 (0.604 - 0.803) | 0.804 | 0.571 | 0.69 | 0.712 | 0.307 | 0.688 | 0.752 |
| Bernoulli NB | 0.704 | 0.760 (0.667 - 0.853) | 0.704 (0.604 - 0.803) | 0.739 | 0.657 | 0.657 | 0.739 | 1 | 0.698 | 0.749 |
| Ensemble RF | 0.807 | 0.759 (0.666 - 0.852) | 0.704 (0.604 - 0.803) | 0.783 | 0.6 | 0.677 | 0.72 | 0.541 | 0.691 | 0.757 |
| Gradient Boosting | 0.755 | 0.758 (0.665 - 0.852) | 0.765 (0.673 - 0.858) | 0.891 | 0.6 | 0.808 | 0.745 | 0.064 | 0.746 | 0.718 |
| Decision Tree | 0.729 | 0.721 (0.624 - 0.819) | 0.679 (0.577 - 0.781) | 0.761 | 0.571 | 0.645 | 0.7 | 0.557 | 0.666 | 0.657 |
| Bagging | 0.789 | 0.720 (0.622 - 0.818) | 0.704 (0.604 - 0.803) | 0.783 | 0.6 | 0.677 | 0.72 | 0.541 | 0.691 | 0.676 |

**RF-Selected Variables (Unbalanced Dataset)**

| Models | Training CV AUC | Testing CV AUC | Testing Accuracy | Testing Sensitivity | Testing Specificity | Testing PPV | Testing NPV | McNemar P-Value | Balanced Accuracy | PR-AUC |
| --- | --- | --- | --- | --- | --- | --- | --- | --- | --- | --- |
| Gaussian NB | 0.834 | 0.793 (0.705 - 0.881) | 0.741 (0.645 - 0.836) | 0.804 | 0.657 | 0.719 | 0.755 | 0.664 | 0.731 | 0.743 |
| Gaussian Process | 0.786 | 0.789 (0.701 - 0.878) | 0.778 (0.687 - 0.868) | 0.826 | 0.714 | 0.758 | 0.792 | 0.815 | 0.77 | 0.749 |
| Ada Boost | 0.849 | 0.789 (0.700 - 0.878) | 0.765 (0.673 - 0.858) | 0.804 | 0.714 | 0.735 | 0.787 | 1 | 0.759 | 0.725 |
| Random Forest | 0.863 | 0.784 (0.694 - 0.873) | 0.765 (0.673 - 0.858) | 0.826 | 0.686 | 0.75 | 0.776 | 0.648 | 0.756 | 0.753 |
| SVM (Linear) | 0.857 | 0.783 (0.693 - 0.873) | 0.704 (0.604 - 0.803) | 0.696 | 0.714 | 0.641 | 0.762 | 0.541 | 0.705 | 0.713 |
| Logistic Regression | 0.796 | 0.780 (0.690 - 0.870) | 0.753 (0.659 - 0.847) | 0.826 | 0.657 | 0.742 | 0.76 | 0.503 | 0.742 | 0.736 |
| Bagging | 0.824 | 0.778 (0.687 - 0.868) | 0.778 (0.687 - 0.868) | 0.826 | 0.714 | 0.758 | 0.792 | 0.815 | 0.77 | 0.711 |
| Ensemble GLM | 0.837 | 0.776 (0.685 - 0.867) | 0.765 (0.673 - 0.858) | 0.826 | 0.686 | 0.75 | 0.776 | 0.648 | 0.756 | 0.724 |
| Ensemble RF | 0.839 | 0.775 (0.684 - 0.866) | 0.704 (0.604 - 0.803) | 0.761 | 0.629 | 0.667 | 0.729 | 0.839 | 0.695 | 0.748 |
| SVM (Radial) | 0.86 | 0.773 (0.682 - 0.864) | 0.728 (0.632 - 0.825) | 0.739 | 0.714 | 0.676 | 0.773 | 0.832 | 0.727 | 0.739 |
| Gradient Boosting | 0.805 | 0.769 (0.677 - 0.861) | 0.753 (0.659 - 0.847) | 0.804 | 0.686 | 0.727 | 0.771 | 0.824 | 0.745 | 0.746 |
| Ensemble GBM | 0.804 | 0.754 (0.661 - 0.848) | 0.704 (0.604 - 0.803) | 0.717 | 0.686 | 0.649 | 0.75 | 0.839 | 0.702 | 0.734 |
| Bernoulli NB | 0.709 | 0.727 (0.630 - 0.824) | 0.691 (0.591 - 0.792) | 0.913 | 0.4 | 0.778 | 0.667 | 0.001 | 0.657 | 0.716 |
| Decision Tree | 0.767 | 0.714 (0.616 - 0.812) | 0.716 (0.618 - 0.814) | 0.761 | 0.657 | 0.676 | 0.745 | 1 | 0.709 | 0.666 |
| KNN | 0.833 | 0.693 (0.592 - 0.793) | 0.580 (0.473 - 0.688) | 0.587 | 0.571 | 0.513 | 0.643 | 0.608 | 0.579 | 0.677 |
