# Supplementary material for: Predicting medication non-adherence using machine learning: Incorporating Complementary and Alternative Medicine (CAM) beliefs in Malaysian chronic disease patients
Source: PLoS One. 2026 Jul 30;21(7):e0354682. doi: 10.1371/journal.pone.0354682 (PMC13423157; doi:10.1371/journal.pone.0354682)
Supplement: S1 File — (DOCX) [file pone.0354682.s003.docx]

**Supplementary File: Detailed description of machine learning aalgorithm and model evaluation analysis.**

**Supplementary Material: Detailed Description of Machine Learning Algorithms**

This study employed twelve machine learning algorithms representing multiple methodological categories. Logistic Regression (LR) and Support Vector Machine (SVM) with a linear kernel were used as linear classifiers suitable for linearly separable data. LR estimates class probabilities using the logistic function, whereas linear SVM identifies the optimal separating hyperplane between classes.

To model more complex relationships, SVM with a radial basis function (RBF) kernel, k-Nearest Neighbours (k-NN), and Gaussian Process were implemented. The RBF kernel maps data into higher-dimensional feature spaces to capture non-linear decision boundaries. k-NN classifies samples according to the majority class among neighbouring observations based on feature similarity. Gaussian Process is a probabilistic non-parametric model capable of estimating prediction uncertainty alongside classification outputs.

Probabilistic methods included Gaussian Naïve Bayes and Bernoulli Naïve Bayes, both derived from Bayes’ theorem under the assumption of conditional independence between variables. Gaussian Naïve Bayes assumes continuous variables follow a Gaussian distribution, whereas Bernoulli Naïve Bayes is designed for binary features.

Tree-based methods included Decision Tree, Random Forest (RF), AdaBoost, Gradient Boosting, and Bagging. Decision Tree models recursively partition the data into subsets using feature-based rules. RF extends this approach by constructing multiple bootstrapped decision trees and aggregating their predictions to reduce variance and overfitting. AdaBoost sequentially trains weak learners by emphasising previously misclassified observations, while Gradient Boosting iteratively minimises prediction errors by fitting new learners to residuals. Bagging improves model stability by aggregating predictions from multiple models trained on bootstrap samples.

To enhance predictive performance, stacked ensemble learning was implemented using all twelve conventional machine learning models as base learners. Their predictions were subsequently combined using three meta-learners: Generalised Linear Model (GLM), Random Forest (RF), and Gradient Boosting Machine (GBM). The Ensemble GLM provided interpretability and linear integration of base learner outputs, Ensemble RF improved robustness through non-linear bagging mechanisms, and Ensemble GBM optimised prediction performance through sequential error correction. This stacked ensemble framework enabled the integration of diverse predictive patterns from heterogeneous algorithms, thereby improving generalisability and classification performance.

**Supplementary Material: Detailed Description of Model Evaluation, Validation, and Interpretation**

Descriptive statistics were used to summarise the study variables, with categorical variables presented as frequencies and continuous variables reported as means and standard deviations. Univariate analyses were conducted to identify variables significantly associated with medication non-adherence. Chi-square tests were applied for categorical variables, while two-sided independent Student’s t-tests were used for continuous variables, with statistical significance defined as p < 0.001.

Predictive model performance was evaluated using multiple classification metrics, including area under the receiver operating characteristic curve (AUC), balanced accuracy, sensitivity, specificity, positive predictive value (PPV), and negative predictive value (NPV). AUC was prioritised as the primary evaluation metric because of its robustness and reduced sensitivity to class imbalance. To statistically compare predictive performance across models, a paired corrected resampled t-test was employed to account for dependencies resulting from repeated resampling and validation procedures.

Following model selection, SHapley Additive exPlanations (SHAP) were used to interpret the best-performing model. SHAP is a model-agnostic interpretability method derived from cooperative game theory that quantifies the contribution of each feature to the prediction output. Positive SHAP values indicated contributions toward predicting non-adherence, whereas negative values indicated contributions toward adherence. Variable importance was ranked using mean absolute SHAP values. Feature importance plots and beeswarm summary plots were generated to visualise the magnitude and direction of variable effects across individual observations.

To establish clinically meaningful classification thresholds, the Youden Index and Decision Curve Analysis (DCA) were performed. The Youden Index identifies the threshold that maximises the sum of sensitivity and specificity, thereby optimising overall classification performance. Although simple and prevalence-independent, the method does not account for differing clinical consequences associated with false positive and false negative predictions.

DCA was additionally conducted to evaluate the net clinical benefit of prediction models across varying probability thresholds. Unlike purely statistical approaches, DCA incorporates the relative clinical consequences of prediction outcomes and compares the model against default strategies such as treating all patients or treating none. This provides insight into the practical utility of the predictive model in clinical decision-making.

Calibration analysis was further performed using Platt scaling to assess whether probability calibration improved model reliability and clinical utility. Comparative evaluation between the original and calibrated models included log loss, Brier score, χ² goodness-of-fit statistics, and associated p-values. All analyses, including preprocessing, statistical testing, performance evaluation, calibration, DCA, and SHAP interpretation, were implemented using Python libraries such as scikit-learn, scipy, and numpy.
